# Multiple psychosocial stressors and coping strategies in relation to sleep health

**DOI:** 10.1093/sleep/zsaf190

**Published:** 2025-07-11

**Authors:** Dana M Alhasan, Frankie LaPorte, Symielle A Gaston, Quaker E Harmon, Anissa I Vines, John A McGrath, W Braxton Jackson II, Luciana Giorgio Cosenzo, Chandra L Jackson

**Affiliations:** Epidemiology Branch, National Institute of Environmental Health Sciences, National Institutes of Health, Department of Health and Human Services, Research Triangle Park, NC, United States; Department of Epidemiology and Community Health, College of Health and Human Services, University of North Carolina at Charlotte, Charlotte, NC, United States; DLH, LLC, Bethesda, MD, United States; Epidemiology Branch, National Institute of Environmental Health Sciences, National Institutes of Health, Department of Health and Human Services, Research Triangle Park, NC, United States; Epidemiology Branch, National Institute of Environmental Health Sciences, National Institutes of Health, Department of Health and Human Services, Research Triangle Park, NC, United States; Department of Epidemiology, University of North Carolina at Chapel Hill, Chapel Hill, NC, United States; DLH, LLC, Bethesda, MD, United States; DLH, LLC, Bethesda, MD, United States; School of Social Work, University of Alabama, Tuscaloosa, AL, United States; Epidemiology Branch, National Institute of Environmental Health Sciences, National Institutes of Health, Department of Health and Human Services, Research Triangle Park, NC, United States; Intramural Program, National Institute on Minority Health and Health Disparities, National Institutes of Health, Department of Health and Human Services, Bethesda, MD, United States

**Keywords:** African Americans, minority groups, sleep, sleep initiation and maintenance disorders, stress, resilience, social support, adaptation

## Abstract

**Study Objectives:**

To determine associations between multiple domains of psychosocial stressors and coping strategies in relation to sleep health among Black/African American (BAA) women.

**Methods:**

Among 1678 BAA participants with complete data enrolled in the Study of Environment, Lifestyle and Fibroids, we conducted principal components analysis on 43 self-reported stressors, yielding six components of psychosocial stressors and three components of coping strategies. Self-reported sleep measures included sleep duration (very short, short, vs. recommended sleep), frequent insomnia symptoms (trouble falling or staying asleep at least 15 days/month vs. <15 days/month), and waking up feeling unrested (≥4 days/week vs. <4 days/week). Using Poisson regression with robust variance while adjusting for sociodemographic characteristics, we estimated prevalence ratios (PRs) and 95% confidence intervals (CIs) for each sleep measure comparing the presence vs. absence of stressors.

**Results:**

Median age (interquartile range) was 29.3 (26.3–32.0) years and 45% had an annual household income of less than $20 000. The following psychosocial stressors were highly prevalent: perceived racism (84.5%), financial strain (84.1%), and emotional distress (73.7%). Emotional distress (PR = 2.26 [95% CI = 1.63 to 3.12]) and financial strain (PR = 1.41 [1.05–1.91]) were associated with more frequent insomnia symptoms. Social/emotional support (PR = 0.80 [0.68–0.95]) and resilience/personal strength (PR = 0.82 [0.70–0.97]) were associated with lower prevalence of very short sleep duration. BAA women reporting experiences with racism vs. not had a higher prevalence of short sleep (PR = 1.14 [1.05–1.23]). Among women who did shift work, financial strain was associated with a 22% (PR = 1.22 [1.03–1.45]) higher prevalence of short sleep.

**Conclusion:**

These findings may inform interventions aimed at addressing stressors associated with poor sleep.

**Statement of Significance**

Based on a novel array of 43 potential psychosocial stressors, we found that stressors such as financial strain, emotional distress, and medical/crime/family problems were associated with poor sleep health (e.g. insomnia symptoms) while coping strategies (e.g. social/emotional support and resilience/personal strength) were associated with more favorable sleep health (e.g. recommended hours of sleep) among an understudied sample of 1678 young Black/African American women. Our study measuring specific, understudied stressors is novel and important to identify which stressors/coping strategies to prioritize for intervention. For instance, our observed associations between racism and medical/crime/family problems in relation to poorer sleep health indicates a need to address medical problems, such as improving healthcare quality, addressing race-based perceived unfair treatment, and increasing access to healthcare.

## Graphical Abstract

**Graphical Abstract:**
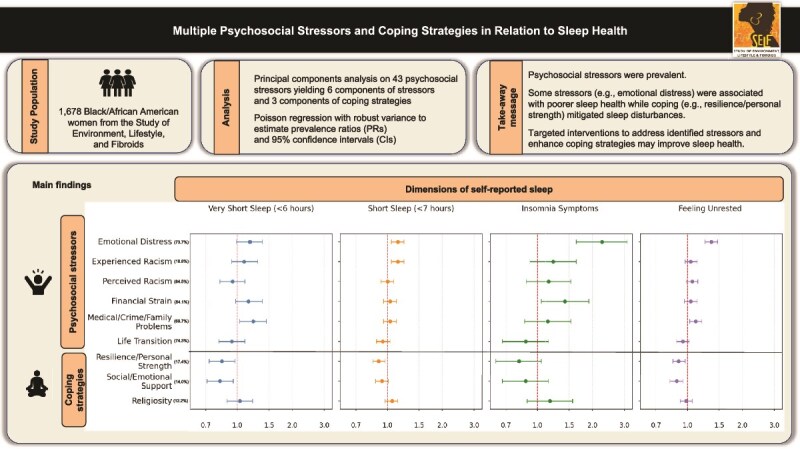

## Introduction

Sleep deficiency and disturbances are highly prevalent,^1^ and they disproportionately impact historically excluded groups in the United States, including women and minoritized racial groups and ethnicities.^2^ Moreover, short sleep duration disparities have widened in the past fifteen years.^3^ Black/African American (BAA) compared to White American adults, generally have shorter sleep duration^4^ and poorer sleep quality.^5^ Women, regardless of race, compared to men are more likely to have insomnia,^6^ and men are more likely to have obstructive sleep apnea.^7^ Since short and disrupted sleep contribute to poor overall health by increasing risk for adverse health outcomes such as obesity, type 2 diabetes, and cardiovascular disease, it is important to identify modifiable determinants of adverse sleep health, especially among disproportionately burdened groups.^8^

Considering both sex and race along with other social determinants of health, BAA women generally have poorer sleep health than White women^9^ and are more likely to experience adverse material (e.g. financial strain) and social (e.g. discrimination based on skin color) conditions.^10^ For instance, racism coupled with sexism (known as misogynoir), can produce unique stressors that may be driving the sleep disparities observed throughout the life course.^11^^,^^12^ Stress may lead to biological alterations that affect sleep health and subsequently lead to adverse health outcomes. For example, arousal of the biological stress response system can activate the sympathetic nervous system and the hypothalamic-pituitary-adrenal (HPA) axis, which can fragment sleep, reduce time spent in slow-wave sleep, and shorten sleeping time.^13^^,^^14^ In fact, psychological stress has been linked to less slow-wave sleep, which is generally considered a physiologically restorative sleep stage.^15^ Stress-related physiological responses likely also contribute to accelerated biological aging or weathering (i.e. allostatic load), which BAA women experience due, at least in part, to intersecting misogynoir.^16^^,^^17^

Furthermore, the adult stressor-sleep relationship could be modified by factors such as adverse childhood factors (CFs), including poverty, and shift work, suggesting the importance of life course theory.^18^ Adverse CFs, such as economic hardship, are more common among BAA compared to other racialized groups of women^19^ and may modify the stressor-sleep relationship by, for instance, damaging brain circuitry during development and fostering maladaptive health behaviors that can be retained into adulthood.^20-22^ Among BAA, early life disadvantage increased the risk of depressive symptoms in adulthood.^23^ Although BAA women experienced the same number of major life events in a year compared to their White counterparts, BAA women reported significantly more financial strain, housing instability, and relationship issues.^24^ Additionally, labor market discrimination^25^ can disproportionately “funnel” BAA women into less desirable jobs and positions that could disproportionately contribute to job stress/strain and, thereby, impact sleep health. Features of the job, such as shift work, could also lead to circadian misalignment, for example.^26^

Given the unique demands on BAA women, BAA women simultaneously rely on positive coping strategies (e.g. resilience/personal strength) to deal with these stressors,^27^ which may buffer the effects of adversity on sleep health.^28^^,^^29^ Yet, overexertion—or a phenomenon known as John Henryism, where strain occurs due to one’s ambition/work ethic or goals not being supported through material and social resources—may also potentially lead to poor health behaviors and outcomes, including poor sleep health.^30^ Therefore, it is important to examine the association between stressors and coping strategies in relation to sleep among BAA women.

While previous studies have demonstrated that psychosocial stressors are linked to poor sleep, few examined this relationship among BAA women.^15^ Among studies including BAA women, few also considered a broad range of potential stressors such as financial strain, medical problems, and job changes, as well as stressors (e.g. racism) more unique to BAA women.^31–33^ Furthermore, previous studies seldom considered the role of coping strategies (e.g. resilience/personal strength). To address these gaps in the prior scientific literature, we investigated multiple domains of psychosocial stressors as well as positive coping strategies in relation to multiple dimensions of sleep health among young BAA women. We hypothesized that psychosocial stressors are associated with poor sleep health while positive coping strategies attenuate the magnitude of the stressor-poor sleep association. We also hypothesized that the magnitude of the stressor-sleep association would be stronger among women with a history of adverse CFs and shift work. Furthermore, we hypothesized that the magnitude of the coping strategies-sleep association would be more protective among women who had no history of adverse CFs and were not shift workers.

## Methods

### Data source: the SELF study

We obtained participant data from the Study of Environment, Lifestyle, and Fibroids (SELF), a prospective cohort study aimed to identify risk factors for uterine fibroid incidence and growth among young BAA women residing in the Detroit, Michigan metropolitan area. Details have been previously described elsewhere.^34^ Briefly, BAA women aged 23–35 years without a clinical diagnosis of fibroids were enrolled from November 2010 to December 2012. At baseline, women self-reported childhood, adolescent, and current sociodemographic and health behavior characteristics and underwent physical examinations (*N* = 1693). We used self-reported data collected at enrollment. All participants provided informed consent, and the SELF protocol was approved by the National Institute of Environmental Health Sciences (NIEHS) Institutional Review Board.

### Study population

We excluded participants with missing data across the 43 psychosocial stressors and coping items (*n* = 15) resulting in a final analytic sample of 1678 women. Differences in sociodemographic and health characteristics among women excluded from the analysis were comparable to those included (Supplemental Table S1).

### Exposure assessment: psychosocial stressors and coping strategies

#### Development of domains of psychosocial stress and coping strategies

We identified domains of psychosocial stressors and coping strategies from 43 self-reported stress items that include the Perceived Stress Scale,^35^ major life events based on the Holmes and Rahe event measures,^36^ financial strain used in previous research in the Pitt County Study,^37^ and previously tested questions on racism (Supplemental Table S2).^38^ We reverse-scored questions, when necessary, to maintain consistent direction across ordinal scales. To reduce the dimensionality of the data, we subjected all of the stress items to principal components analysis (PCA) of their polychoric correlation matrix. We considered extracts of 6–12 components and determined nine components, accounting for 57 per cent of the total variance (Supplemental Table S3), to be the best solution based on component loadings of at least 0.3^^39^^ and scree plots. Our choice of nine components was confirmed by Velicer’s minimum average partial (MAP) test as well as parallel analysis.^40-42^ The MAP test relies on partial correlations to estimate common variance within the data, while parallel analysis compares the variance explained by the components to that of randomly generated components. Each participant then received a score for each of the nine components by summing their standardized responses multiplied by the component loadings.^43^ These component scores were then dichotomized at the median into “high” and “low” scores and used in subsequent analyses.^44^^,^^45^ Since the components are centered at zero, this effectively labels scores above zero as the “high” component scores and those below zero as the “low” component scores. We relabeled the nine components based on their apparent domain, which included six psychosocial stressor components (1) emotional distress, (2) experienced racism, (3) perceived racism; (4) financial strain, (5) medical/crime/family problems, and (6) life transitions as well as three coping strategy components (1) resilience/personal strength, (2) social/emotional support, and (3) religiosity.

#### Psychosocial stressors

For psychosocial stressors, higher values indicated a higher number of stressors in the given domain. Items assessed general frequency or specified a range (e.g. past 30 days). Emotional distress included frequency of feeling the need to suppress or swallow strong feelings of anger, frequency of finding yourself several days later mentally replaying conversations or events that did not go your way, frequency of feeling unable to control important things in life in the past 30 days, frequency of yelling/shouting at yourself or someone else to let off steam, frequency of keeping hurt feelings to yourself, frequency of feeling difficulties were piling so high that you could not overcome them in the past 30 days, day-to-day life stress level in the past 12 months, and having difficulties with your current significant other or spouse in the past 12 months. Experienced racism included frequency experienced racism during 20s, frequency experienced racism before age 20, experienced racism/discrimination in past 12 months, frequency White indivduals used slurs against you in the past 5 years, and frequency thought about own race. Perceived racism included general frequency Black adults watched more closely as they shop than White adults, frequency felt being watched due to race in past 5 years, and Black adults still subject to slurs from White adults. Financial strain included difficulty in the past 12 months related to housing, affording food/clothes, falling behind on bills, moving to new home, losing a job or fearing job loss, and difficulty paying basic expenses (food, clothing, shelter, medical care, transportation). Medical/crime/family problems included the following in the past 12 months: major medical problem, major problems with kids, someone close had major medical problem, victim of violent crime, problems with other family, experienced other major change/event, and victim of non-violent crime. Life transitions included the following in the past 12 months: new romantic relationship, difficulties with former spouse or partner, and started new job.

#### Coping strategies

For these three sets of components, higher values indicated more support in the given domain. Resilience/personal strength included frequency of feeling confident about ability to handle personal problems in the past 30 days, frequency of feeling things going your way in the past 30 days, and frequency of talking over a problem and finding a fair compromise. Social/emotional support included family believed in/supported as a child, family made participant feel special as a child, can count on someone for help, can count on someone for emotional support, and number of people feel close to. Religiosity included amount religion/spirituality is source of strength/comfort, importance of faith, and frequency of prayer/meditation.

### Outcome assessment: sleep health

Sleep duration, measured as the number of hours participants slept within a 24-hour period, was categorized as very short (<6 hours), short (<7 hours), and recommended (7–9 hours; reference group), based on the recommendations from the National Sleep Foundation, which also allowed us to capture long sleep^46^ as opposed to the SRS/AASM joint recommendations to dichotomize sleep duration.^47^ Very short and short sleep were not mutually exclusive. Long sleep (>9 hours) had a small sample size (*n* = 18), and these participants were excluded from the analyses of sleep duration but were included in the analyses of other sleep measures. Frequent insomnia symptoms were defined as trouble falling or staying asleep at least 15 days per month vs. fewer than 15 days per month. Frequently waking up feeling unrested was defined as at least 4 days per week versus fewer than 4 days. These cut-off points allow us to capture the most affected individuals who experienced the symptoms for at least half the month and woke up feeling unrested for over half the week. Finally, we created a total sleep score where the following were assigned one point each for: very short, short, or long sleep duration (where very short and short sleep were mutually exclusive); frequent insomnia symptoms; and waking up feeling unrested. The total poor sleep score ranged from 0 to 3, with a higher score indicating poorer sleep.

### Potential confounders

Based on literature, we considered, a priori, the following confounders: age (measured continuously and analyzed categorically in some analyses), employment status (not employed, <30 hours per week, and ≥30 hours per week), educational attainment (high school or equivalent [e.g. General Educational Development, GED] or less; some college, Associates or Technical degree; and Bachelors or higher degree), marital status (currently married or living as married, formerly married, or single), annual household income (<$20 000, $20 000–50 000, or ≥$50 000), smoking status (former, current, never), alcohol consumption in the past 12 months (none, moderate, or heavy), work and leisure physical activity (low, low to moderate, moderate, high and very high), asthma, cardiovascular risk (defined below), and mental health diagnoses (defined below). Cardiovascular disease risk was defined as “higher” if the participant reported at least one of the following diagnoses: hypertension, high cholesterol, diabetes, heart attack, angina, stroke, or body mass index (BMI) of at least 25 kg/m^2^ based on measured weight and height.^48^ “Lower” cardiovascular risk was defined as the absence of all the aforementioned conditions. Mental health diagnoses (yes/no) were defined as self-report of ever being diagnosed with depression, bipolar disorder, anxiety, panic, and/or post-traumatic stress disorder (PTSD).

### Potential modifiers: CFs and worked any rotating shifts

Adverse CFs were based on three childhood variables: sleep, neighborhood safety, and resources (e.g. enough to eat). Childhood sleep was categorized as poor sleep (yes) if participants reported any of the following as “never” or “rarely”: (1) “in bed by 8 pm”, (2) “slept in dark room or only night light,” or (3) “slept in quiet room”. A separate childhood sleep variable was light sleeper (yes/no) based on the following question, “When you were around 5 years old, were you a light sleeper, that is easily awakened?” Participants rated the safety of their neighborhood at 5, 10, and 15 years of age as unsafe, somewhat safe, or very safe. Neighborhood safety was categorized as safe (if all responses indicated safe locations), somewhat safe (if there was a mix of safe and unsafe responses), and unsafe (if there were no safe responses for all three questions at ages 5, 10, and 15). In terms of childhood resources, participants were categorized as “poor” if they both (1) marked yes to “when you were growing up, were there times when you didn’t have enough to eat” and (2) answered low-income to “household income while growing up”. In sensitivity analyses, we evaluated each adverse CF individually (i.e. childhood sleep, neighborhood safety, and resources) as well as childhood light sleep. An additional modifier was worked for any rotating shifts (predictable shifts day-to-day) (yes versus no).

### Statistical analyses

**Figure 1:**
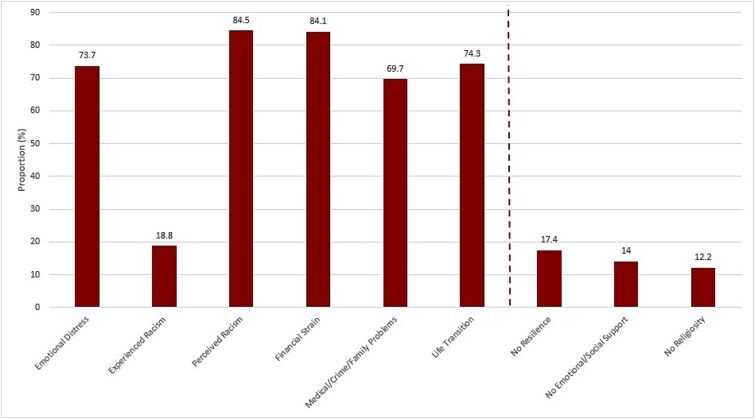
Prevalence of stressors and poor coping strategies characteristics, SELF, 2010–2012 (*N* = 1678). Participants were counted as having the stress/poor coping strategy if they selected one of the highest (top two) responses from one or more Likert items from that scale.

We computed descriptive statistics and presented continuous variables as median and interquartile range (IQR) and categorical variables as frequencies and percentages. To calculate the prevalence of each component identified by the PCA, participants were counted as having the stress/poor coping strategy if they selected one of the top two highest responses from one or more of the Likert items associated with the component (Figure 1). We also show sociodemographic characteristics by low compared to high stressors and coping strategies (Supplemental Table S4). We conducted Poisson regression with robust variance to estimate prevalence ratios (PRs) and 95 percent confidence intervals (CIs) to evaluate the association between each of the psychosocial stressors and coping strategies separately with categorical sleep health outcomes (yes/no) or total sleep score (count variable). All models had three progressive levels of adjustment: model 1 adjusted for age, employment status, educational attainment, marital status, and annual household income; model 2 additionally adjusted for cardiovascular risk, mental health diagnoses, and asthma; and model 3 additionally adjusted for smoking status, alcohol consumption, and physical activity level. Effect modification by adverse CFs (yes vs. no) and worked any rotating shifts (yes vs. no) was tested with the inclusion of interaction terms with each psychosocial stressor and coping strategy as a cross product in the fully adjusted models (e.g. adverse CFs*experienced racism). In supplemental analyses, we examined each component of CF (i.e. sleep, neighborhood safety, and resources) as modifiers between psychosocial stressors/coping strategies and sleep health (Supplemental Tables S5–S8). We used SAS version 9.4 for Windows (Cary, North Carolina) to conduct analyses and determined statistical significance using a two-sided *p*-value of .05.

## Results

### Study population characteristics

Among 1678 women, median age (IQR) of study participants was 29.3 (26.3–32.1) years, most (59.4%) had BMI of 30 kg/m^2^ or higher, and 45.4% earned less than $20 000 annually (Table 1). While 26.6% of participants reported very short sleep and 32% reported short sleep, 62.1% reported waking up feeling unrested (Table 1). In order of contribution to explaining the variance, components included “emotional distress”, “social/emotional support”, “experienced racism,” “financial strain”, “medical/crime/family problems,” “religiosity,” “resilience/personal strength,” “life transitions”, and “perceived racism” (Supplemental Table 3). Many women reported perceived racism (84.5%), financial strain (84.1%), and emotional distress (73.7%) (Figure 1).

**Table 1:** Characteristics of the SELF participants

Characteristics		Study sample (*N* = 1678)
Age, years	Median (IQR)	29.3 (26.3–32.1)
Annual household income	<$20 000	757 (45.4%)
	$20 000–50 000	626 (37.6%)
	>$50 000	284 (17.0%)
Education attainment	High school/GED or less	359 (21.4%)
	Some college/associates/technical	847 (50.5%)
	Bachelors/masters/PhD	472 (28.1%)
Employment	Not employed	635 (37.8%)
	<30 hours	209 (12.5%)
	≥30 hours	834 (49.7%)
Works rotating shifts	Yes	289 (17.2%)
	No	1389 (82.8%)
Marital status	Currently married or living as married	463 (27.6%)
	Formerly married or living as married	236 (14.1%)
	Single	979 (58.3%)
Smoking status	Never	1233 (73.5%)
	Former	124 (7.4%)
	Current	321 (19.1%)
Alcohol consumption	None	438 (26.1%)
	Moderate	551 (32.8%)
	Heavy	689 (41.1%)
Physical activity	Low	264 (15.8%)
	Low-to-moderate	382 (22.8%)
	Moderate	389 (23.3%)
	High	299 (17.9%)
	Very high	339 (20.3%)
Mental health diagnosis	Yes	325 (19.6%)
	No	1336 (80.4%)
BMI	<25 kg/m^2^	333 (19.8%)
	25–29 kg/m^2^	349 (20.8%)
	≥30 kg/m^2^	996 (59.4%)
Cardiovascular risk	Lower risk	307 (18.4%)
	Higher risk	1363 (81.6%)
Asthma	Yes	327 (19.7%)
	No	1334 (80.3%)
Childhood sleep: age 5 in bed by 8	Rarely or never	153 (9.1%)
	Sometimes	372 (22.2%)
	Often	400 (23.9%)
	Most of the time or always	752 (44.8%)
Childhood sleep: in a quiet room	Rarely or never	79 (4.7%)
	Sometimes	209 (12.5%)
	Often	379 (22.6%)
	Most of the time or always	1011 (60.3%)
Childhood sleep: unlit or night light	Rarely or never	183 (10.9%)
	Sometimes	240 (14.3%)
	Often	320 (19.1%)
	Most of the time or always	935 (55.7%)
Childhood light sleeper	Yes	557 (33.2%)
	No	1120 (66.8%)
Childhood sleep composite	Poor sleep	779 (46.4%)
	Good sleep	899 (53.6%)
Neighborhood safety at age 5	Very unsafe	92 (5.5%)
	Somewhat unsafe	248 (14.8%)
	Somewhat safe	691 (41.2%)
	Very safe	647 (38.6%)
Neighborhood safety at age 10	Very unsafe	89 (5.3%)
	Somewhat unsafe	279 (16.6%)
	Somewhat safe	738 (44.0%)
	Very safe	572 (34.1%)
Neighborhood safety at age 15	Very unsafe	137 (8.2%)
	Somewhat unsafe	387 (23.1%)
	Somewhat safe	707 (42.1%)
	Very safe	447 (26.6%)
Neighborhood safety composite	Unsafe	680 (40.5%)
	Safe	998 (59.5%)
Childhood income	High Income	124 (7.4%)
	Middle Income	883 (52.7%)
	Low Income	590 (35.2%)
	Poor	80 (4.8%)
Childhood food insecurity	Yes	215 (12.8%)
	No	1462 (87.2%)
Childhood economic composite	Inadequate resources	715 (42.6%)
	Adequate resources	962 (57.4%)
Sleep duration	Very Short (<6)	446 (26.6%)
	Short (<7)	537 (32.0%)
	Recommended (7–9)	677 (40.3%)
	Long (≥10)	18 (1.1%)
Wake not rested	Yes	1042 (62.1%)
	No	636 (37.9%)
Insomnia symptoms	Yes	175 (10.4%)
	No	1503 (89.6%)
Sleep score	0 (Ideal)	94 (5.6%)
	1	612 (36.5%)
	2	744 (44.3%)
	3 (Poor)	228 (13.6%)

Very short and short sleep are mutually exclusive.

Total sleep score was calculated as one point for each of the following: very short, short, or long sleep duration (where very short and short sleep were mutually exclusive); frequent insomnia symptoms; and waking up feeling unrested. Poor sleep score ranged from 0 to 3 with a higher score indicating poorer sleep.

We excluded participants with missing data on psychosocial stressors and coping strategies (*n* = 15) resulting in a final analytic sample of 1678 women.

The following characteristics had missing variables: annual household income (*n* = 11), physical activity (*n* = 5), mental health diagnoses (*n* = 17), cardiovascular risk (*n* = 8), asthma (n = 17), childhood sleep by age 5 in bed by 8 (*n* = 1), childhood light sleeper (*n* = 1), childhood income (*n* = 1), childhood food insecurity (*n* = 1).

The prevalence of a number of sociodemographic characteristics was different in BAA women with high compared to low coping strategies (Supplemental Table 4). For instance, those with higher resilience or social/emotional support were more likely to have an income of at least $50 000 (18.3%, 22.1%, respectively) compared to those with lower resilience or social/emotional support (15.8%, 12.0%, respectively). Likewise, BAA women with higher religiosity, resilience or social/emotional support were more likely to have at least a bachelor’s degree or more (31.0%, 29.8%, 35.9%, respectively) compared to those with lower religiosity, resilience, or social/emotional support (25.3%, 26.5%, and 20.4%, respectively). Finally, more BAA women smoked (24.7%) among the low social/emotional support group compared to the high social/emotional support (13.6%).

### Psychosocial stressors and coping strategies in relation to sleep

Since model results did not differ between models 1, 2, and 3, we present results from the fully adjusted model (i.e. model 3). In the fully adjusted model, higher compared to lower emotional distress (PR = 2.26 [95% CI = 1.63 to 3.12]) and financial strain (PR = 1.41 [1.05–1.91]) were associated with insomnia symptoms (Table 2). More social/emotional support (PR = 0.80 [0.68–0.95]) and resilience/personal strength (PR = 0.82 [0.70–0.97]) were associated with lower prevalence of very short sleep duration and lower prevalence of days waking up feeling unrested (PR = 0.87 [0.80–0.94] and PR = 0.89 [0.83–0.97], respectively). Higher resilience/personal strength was associated with 11% lower prevalence of short sleep (PR = 0.89 [0.83–0.97]). More personal experience of racism was associated with an increase in poor sleep measures: short sleep (PR = 1.14 [1.05–1.23]) and overall poor sleep score (PR = 1.07 [1.02–1.12]). Similarly, more medical/crime/family problems were associated with 22% higher prevalence of very short sleep (PR = 1.22 [1.03–1.44]) and 11% higher prevalence of waking up feeling unrested (PR = 1.11 [1.03–1.20]) (Table 2).

**Table 2:** PRs of sleep health by stressors and coping strategies, SELF

2010–2012 (*N* = 1661)	Very short sleep (<6 hours) vs. 7–9 hours	Short sleep (<7 hours) vs. 7–9 hours	Frequent insomnia symptoms ^*^ (≥15 days per month)	Wake up feeling unrested (≥4 days per week)	Total sleep score ^†^
Emotional distress	1.17 (0.99, 1.37)	**1.14** **(1.05, 1.23)**	**2.26** **(1.63, 3.12)**	**1.35** **(1.25, 1.46)**	1.02 (0.97, 1.07)
					
Experienced racism	1.09 (0.93, 1.29)	**1.14** **(1.05, 1.23)**	1.22 (0.91, 1.63)	1.04 (0.97, 1.13)	**1.07** **(1.02, 1.12)**
Perceived racism	0.94 (0.80, 1.10)	1.00 (0.92, 1.08)	1.15 (0.87, 1.52)	1.06 (0.98, 1.14)	0.99 (0.95, 1.04)
Financial strain	1.15 (0.98, 1.37)	1.03 (0.95, 1.12)	**1.41** **(1.05, 1.91)**	1.04 (0.96, 1.13)	1.01 (0.96, 1.06)
Medical/crime/family problems	**1.22** **(1.03, 1.44)**	1.03 (0.95, 1.12)	1.14 (0.85, 1.52)	**1.11** **(1.03, 1.20)**	1.01 (0.96, 1.06)
Life transitions	0.93 (0.79, 1.10)	0.94 (0.87, 1.03)	0.86 (0.64, 1.15)	0.94 (0.87, 1.02)	1.00 (0.95, 1.04)
Resilience/personal strength	**0.82** **(0.70, 0.97)**	**0.89** **(0.83, 0.97)**	0.79 (0.59, 1.05)	**0.89** **(0.83, 0.97)**	0.98 (0.94, 1.03)
Social/emotional support	**0.80** **(0.68, 0.95)**	0.93 (0.86, 1.01)	0.86 (0.64, 1.15)	**0.87** **(0.80, 0.94)**	0.99 (0.95, 1.04)
Religiosity	1.03 (0.88, 1.21)	1.06 (0.97, 1.14)	1.17 (0.88, 1.56)	0.98 (0.91, 1.06)	1.03 (0.99, 1.08)
PR = prevalence ratio; CI = confidence interval.					
Model adjusted for age (measured continuously), employment status, educational attainment, marital status, annual household income, cardiovascular risk, mental health diagnoses, asthma, smoking status, alcohol consumption, and physical activity. Bolded estimates reflect statistical significance of a likelihood ratio test at a two-sided p < 0.05.					
^*^Frequent insomnia symptoms defined as either trouble falling asleep or waking up during the night at least 15 days a month.					
^†^Higher total sleep score indicates poorer sleep. Total sleep score was calculated as one point for each of the following: very short, short, or long sleep duration (where very short and short sleep were mutually exclusive); frequent insomnia symptoms; and waking up feeling unrested. The total poor sleep score ranged from 0 to 3, with a higher score indicating poorer sleep.					

### Psychosocial stressors, coping strategies, and sleep by childhood life factors

The association between social/emotional support and short sleep duration differed by CFs (*p*-value for effect modification = .0016). Higher social emotional support was associated with a reduced prevalence of very short sleep (PR = 0.47 [0.33–0.69] among women with favorable CFs, while those with adverse CFs had no association (PR = 0.93 [0.78–1.12]) (Table 3). The association between emotional stress and waking unrested differed by CFs (*p*-value = .017), such that higher emotional distress was associated with a higher prevalence of waking unrested among both groups; however, the PRs was larger among those with favorable CFs (PR = 1.63 [1.35–1.97]) compared to those with adverse CFs (PR = 1.28 [1.17–1.39]). The association between life transitions and very short sleep duration differed by CFs (*p*-value for effect modification = .022). Among women with adverse CFs, higher life transition stress was associated with a 15% lower prevalence of very short sleep (PR = 0.85 [0.71–1.02]) while those with favorable CFs and higher compared to lower life transition stress had a 40% higher prevalence of very short sleep (PR = 1.40 [0.94–2.06]). Total sleep score associations with higher life transition stress also differed by CF status (*p*-value for effect modification = .031). Those with adverse CF and higher life transition stress had a small reduction in the overall sleep score (PR = 0.97 [0.92–1.02]) while those with favorable CFs and higher life transition stress had a small increase in the overall sleep score (PR = 1.09 [0.99–1.20]).

**Table 3:** PRs of sleep health by stressors and coping strategies stratified by childhood life factors, SELF, 2010–2012 (*N* = 1661)

**Sleep outcomes**	**Very short sleep** **(<6 hours)** **vs. 7**–**9 hours**		**Short sleep** **(<7 hours)** **vs. 7**–**9 hours**		**Frequent insomnia** ^*^ **symptoms** **(≥15 days per month**)		**Wake up feeling unrested** **(≥4 days per week)**		**Total sleep score** ^**†**^	
	**PR (95% CI)**	**PR (95% CI)**	**PR (95% CI)**	**PR (95% CI)**	**PR (95% CI)**	**PR (95% CI)**	**PR (95% CI)**	**PR (95% CI)**	**PR (95% CI)**	**PR (95% CI)**
**Childhood life factors**	**Adverse**	**Favorable**	**Adverse**	**Favorable**	**Adverse**	**Favorable**	**Adverse**	**Favorable**	**Adverse**	**Favorable**
Emotional distress	1.10 (0.92, 1.31)	1.49 (1.02, 2.18)	1.14 (1.04, 1.25)	1.12 (0.93, 1.34)	2.07 (1.45, 2.94)	3.22 (1.50, 6.91)	**1.28** **(1.17, 1.39)**	**1.63** **(1.35, 1.97)**	1.03 (0.97, 1.08)	0.99 (0.91, 1.09)
Experienced racism	1.15 (0.96, 1.38)	0.84 (0.58, 1.23)	1.12 (1.02, 1.22)	1.19 (0.98, 1.43)	1.10 (0.80, 1.50)	1.95 (0.96, 3.96)	1.05 (0.97, 1.14)	0.99 (0.82, 1.19)	1.08 (1.02, 1.14)	1.05 (0.96, 1.16)
Perceived racism	0.89 (0.74, 1.05)	1.16 (0.79, 1.69)	1.00 (0.91, 1.09)	1.01 (0.84, 1.21)	1.16 (0.86, 1.58)	1.07 (0.55, 2.10)	1.03 (0.95, 1.12)	1.12 (0.94, 1.35)	1.01 (0.96, 1.06)	0.94 (0.85, 1.03)
Financial strain	1.18 (0.98, 1.41)	1.06 (0.72, 1.56)	1.07 (0.97, 1.17)	0.90 (0.75, 1.09)	1.63 (1.16, 2.28)	0.77 (0.39, 1.52)	1.05 (0.96, 1.14)	1.02 (0.85, 1.23)	1.00 (0.95, 1.06)	1.04 (0.95, 1.15)
Medical/crime/family problems	1.17 (0.98, 1.40)	1.50 (1.01, 2.24)	1.03 (0.94, 1.13)	1.03 (0.85, 1.24)	1.04 (0.76, 1.41)	1.93 (0.90, 4.14)	1.11 (1.03, 1.21)	1.14 (0.95, 1.38)	1.01 (0.96, 1.07)	0.99 (0.90, 1.09)
Life transitions	**0.85** **(0.71, 1.02)**	**1.40** **(0.94, 2.06)**	0.92 (0.84, 1.01)	1.06 (0.88, 1.28)	0.78 (0.57, 1.07)	1.39 (0.70, 2.76)	0.96 (0.88, 1.05)	0.87 (0.72, 1.05)	**0.97** **(0.92, 1.02)**	**1.09** **(0.99, 1.20)**
Resilience/personal strength	0.82 (0.69, 0.98)	0.84 (0.57, 1.22)	0.93 (0.85, 1.02)	0.77 (0.64, 0.92)	0.77 (0.56, 1.06)	0.89 (0.46, 1.74)	0.93 (0.86, 1.01)	0.77 (0.64, 0.92)	0.99 (0.94, 1.05)	0.96 (0.87, 1.05)
Social/emotional support	**0.93** **(0.78, 1.12)**	**0.47** **(0.33, 0.69)**	0.95 (0.87, 1.05)	0.90 (0.74, 1.08)	0.98 (0.71, 1.34)	0.55 (0.28, 1.07)	0.91 (0.83, 0.99)	0.79 (0.66, 0.95)	0.99 (0.94, 1.05)	1.01 (0.91, 1.11)
Religiosity	1.08 (0.91, 1.29)	0.83 (0.57, 1.21)	1.04 (0.95, 1.14)	1.10 (0.92, 1.33)	1.08 (0.79, 1.48)	1.65 (0.82, 3.31)	0.95 (0.87, 1.03)	1.10 (0.91, 1.32)	1.01 (0.96, 1.07)	1.10 (1.00, 1.21)

PR = prevalence ratio; CI = confidence interval.

Model adjusted for age (measured continuously), employment status, educational attainment, marital status, annual household income, cardiovascular risk, mental health diagnoses, asthma, smoking status, alcohol consumption, and physical activity.

Bolded estimates represent significance at *p* < 0.05 for the type 3 test of the interaction term.

^*^Frequent insomnia symptoms defined as either trouble falling asleep or waking up during the night, at least 15 days a month.

^†^Higher total sleep score indicates poorer sleep. Total sleep score was calculated as one point for each of the following: very short, short, or long sleep duration (where very short and short sleep were mutually exclusive); frequent insomnia symptoms; and waking up feeling unrested. The total poor sleep score ranged from 0 to 3 with a higher score indicating poorer sleep.

Childhood life factors was based on one or more affirmative responses based on childhood sleep, childhood neighborhood safety, and childhood resources. Childhood sleep was measured as poor sleep if participants marked any of the following as “never” or “rarely”: (1) “in bed by 8 pm”, (2) “dark room/ night light” and (3) “quiet room”. Participants rated the safety of their neighborhood at 5, 10, and 15 years of age as unsafe, somewhat safe, or very safe. Neighborhood safety was measured as safe (if all responses indicated safe locations), somewhat safe (if there was a mix of safe and unsafe responses) and unsafe (if there were no safe responses for all three questions at ages 5, 10, and 15). Childhood resources was measured as poor if participants (1) marked yes to “ever not enough to eat” and (2) answered low income to “household income while growing up”.

### Psychosocial stressors, coping strategies, and sleep by shift work status

Among participants who did rotating shift work, participants with higher compared to lower financial strain had a 22% higher prevalence of short sleep (PR = 1.22 [1.03–1.45]) while there was no association among participants without shift work (PR = 0.99 [0.90–1.09]) (Table 4).

**Table 4:** PRs of sleep health by stressors and coping strategies stratified by reports of shift work status, SELF, 2010–2012 (*N* = 1661)

**Sleep outcomes**	**Very short sleep** **(<6 hours)** **vs. 7–9 hours**		**Short sleep** **(<7 hours)** **vs. 7–9 hours**		**Frequent insomnia** ^*^ **symptoms** **(≥15 days per month**		**Wake up feeling unrested** **(≥4 days per week)**		**Total sleep score** ^**†**^	
	**PR (95% CI)**	**PR (95% CI)**	**PR (95% CI)**	**PR (95% CI)**	**PR (95% CI)**	**PR (95% CI)**	**PR (95% CI)**	**PR (95% CI)**	**PR (95% CI)**	**PR (95% CI)**
**Shift work**	**Yes**	**No**	**Yes**	**No**	**Yes**	**No**	**Yes**	**No**	**Yes**	**No**
Emotional distress	1.27 (0.87, 1.84)	1.15 (0.96, 1.37)	1.14 (0.97, 1.35)	1.14 (1.04, 1.25)	2.68 (1.10, 6.51)	2.20 (1.56, 3.10)	1.25 (1.03, 1.52)	1.37 (1.26, 1.49)	1.08 (0.97, 1.20)	1.01 (0.96, 1.06)
Experienced racism	1.00 (0.69, 1.44)	1.12 (0.93, 1.34)	1.07 (0.90, 1.26)	1.15 (1.05, 1.26)	1.87 (0.82, 4.30)	1.14 (0.84, 1.55)	1.15 (0.95, 1.40)	1.03 (0.94, 1.12)	1.02 (0.91, 1.13)	1.09 (1.03, 1.14)
Perceived racism	0.79 (0.54, 1.15)	0.97 (0.81, 1.16)	0.87 (0.73, 1.03)	1.04 (0.95, 1.14)	1.19 (0.56, 2.53)	1.14 (0.84, 1.54)	0.99 (0.82, 1.20)	1.07 (0.98, 1.16)	0.97 (0.88, 1.08)	1.00 (0.95, 1.05)
Financial strain	1.52 (1.02, 2.25)	1.09 (0.91, 1.31)	**1.22** **(1.03, 1.45)**	0.99 (0.90, 1.09)	0.86 (0.40, 1.85)	1.55 (1.12, 2.15)	1.14 (0.93, 1.38)	1.03 (0.94, 1.12)	0.99 (0.89, 1.10)	1.01 (0.96, 1.07)
Medical/crime/family problems	1.23 (0.85, 1.77)	1.22 (1.01, 1.46)	1.10 (0.94, 1.29)	1.01 (0.92, 1.11)	1.10 (0.52, 2.32)	1.14 (0.83, 1.57)	1.11 (0.92, 1.34)	1.11 (1.02, 1.21)	1.03 (0.93, 1.15)	1.00 (0.95, 1.06)
Life transitions	0.80 (0.55, 1.15)	0.96 (0.80, 1.15)	0.86 (0.74, 1.01)	0.97 (0.88, 1.06)	1.25 (0.57, 2.74)	0.80 (0.58, 1.10)	0.89 (0.74, 1.08)	0.95 (0.87, 1.03)	0.92 (0.83, 1.02)	1.01 (0.96, 1.07)
Resilience/personal strength	0.69 (0.47, 1.01)	0.85 (0.71, 1.02)	0.88 (0.75, 1.03)	0.90 (0.82, 0.99)	0.42 (0.18, 0.98)	0.87 (0.64, 1.18)	0.88 (0.73, 1.07)	0.90 (0.82, 0.97)	1.04 (0.94, 1.16)	0.97 (0.93, 1.02)
Social/emotional support	0.89 (0.61, 1.30)	0.79 (0.65, 0.94)	1.02 (0.86, 1.20)	0.91 (0.83, 1.00)	1.76 (0.82, 3.77)	0.76 (0.56, 1.04)	0.84 (0.69, 1.02)	0.87 (0.80, 0.95)	1.01 (0.91, 1.12)	0.99 (0.94, 1.04)
Religiosity	1.17 (0.81, 1.69)	1.01 (0.84, 1.20)	1.07 (0.91, 1.25)	1.05 (0.96, 1.15)	0.98 (0.46, 2.10)	1.21 (0.89, 1.65)	0.97 (0.80, 1.17)	0.98 (0.90, 1.06)	1.08 (0.97, 1.19)	1.02 (0.97, 1.08)

PR = prevalence ratio; CI = confidence interval.

Model adjusted for age (measured continuously), employment status, educational attainment, marital status, annual household income, cardiovascular risk, mental health diagnoses, asthma, smoking status, alcohol consumption, and physical activity.

Bolded estimates represent significance at *p* < 0.05 for the type 3 test of the interaction term.

^*^Frequent insomnia symptoms defined as either trouble falling asleep or waking up during the night, at least 15 days a month.

^†^Higher total sleep score indicates poorer sleep. Total sleep score was calculated as one point for each of the following: very short, short, or long sleep duration (where very short and short sleep were mutually exclusive); frequent insomnia symptoms; and waking up feeling unrested. The total poor sleep score ranged from 0 to 3 with a higher score indicating poorer sleep.

Cardiovascular risk was defined as low if participants have none of the following: hypertension, high cholesterol, diabetes, heart attack, angina, stroke, BMI more than 24 kg/m^2^.

## Discussion

Among a cohort of young BAA women, we identified the domains of “experienced racism,” “perceived racism,” “emotional distress,” “financial strain,” “medical/crime/family problems,” and “life transitions” as psychosocial stressors along with “social/emotional support,” “religiosity,” and “resilience/personal strength” as coping strategies. Psychosocial stressors were common in the study sample, with the perceived racism domain having the highest prevalence (84.5%). We also found that psychosocial stressors were generally associated with poorer sleep health, while positive coping strategies were associated with more favorable sleep. Specifically, emotional distress was strongly associated with short sleep, insomnia symptoms, and feeling unrested. Financial strain was also associated with insomnia symptoms, and medical/crime/family problems were associated with very short sleep and feeling unrested. Meanwhile, social/emotional support and resilience/personal strength were associated with more favorable sleep, including a lower prevalence of feeling unrested and a higher prevalence of attaining the recommended hours of sleep. Having adverse CFs and shift work modified some of the associations between psychosocial stressors as well as between coping strategies and sleep health. Unexpectedly, associations between stress (emotional distress and life transitions) and sleep appear stronger among participants without adverse CFs. However, social/emotional support had a stronger protective association against very short sleep in those with favorable CFs than adverse CFs, suggesting the effect of childhood experiences on adult sleep may be complex. The associations between stress/coping strategies and sleep were largely similar among those who report rotating shift work vs. those who do not. However, a higher prevalence of short sleep was observed due to high financial strain in those reporting shift work, where a non-significant association was observed in those who did not report shift work.

Our finding that psychosocial stressors are associated with poorer sleep health is similar to previous findings. For instance, a systematic review found that 17 studies identified at least one association between discrimination (e.g. racial everyday discrimination) and a measure of poor sleep health (e.g. shorter sleep duration).^49^ A previous study using the Jackson Heart Study cohort found that global perceived stress was associated with higher odds of shorter sleep and poorer sleep quality among BAA participants.^31^ Another study reported that participants, including BAA women, who experienced both everyday and major racial/ethnic discrimination vs. neither reported shorter sleep duration and more insomnia symptoms.^50^ Similarly, a cohort study of BAA women reported that gendered racial microaggression (e.g. feeling silenced and marginalized) was associated with poor sleep quality.^51^ Further, another study using nationally representative data found that associations between financial hardship and sleep disturbances were strongest among BAA participants.^52^

We extended beyond the current literature by demonstrating effect modification by rotating shift work.^53^ Specifically, among shift workers, BAA women with more compared to less financial strain had shorter sleep duration. However, financial strain was not associated with short sleep duration among women who reported no shift work. A systematic review noted effectiveness of non-pharmacological interventions for chronic disease risk factors, including sleep health, among shift workers, including schedule change, behavioral change, and controlled light exposure.^54^ Therefore, organizations and workplaces can implement interventions, such as two-shift schedules, that may improve sleep health outcomes among shift workers. The finding that relationships between higher compared to lower emotional distress and frequently waking feeling unrested were stronger among BAA women with favorable (rather than adverse) CFs is unexpected. It is well documented that adverse CFs increase risk of future poor health (e.g. cardiovascular health) through behaviors, such as poor sleep.^55^ A systematic review of 30 studies reported adverse childhood experiences (ACEs) to be associated with poor sleep among women.^56^ It is possible that women with adverse CFs have poorer sleep in general, which would attenuate the relative associations with adulthood life stressors in the group with adverse CFs in our cross-sectional study. In other words, the likely higher prevalence of poor sleep in childhood and adolescence of individuals with ACEs could make it more difficult to observe a statistically significant relative association related to the additional life stressors in adulthood. More longitudinal, life course studies and investigations of mediation are warranted. The impact of cumulative exposures, including those early in life, demonstrates how the “wear and tear” on the body and allostatic load may impact health later in life. Therefore, early life conditions set the stage for health later in life, although we do not always account for them, indicating the need to intervene on societal conditions early in life to ensure favorable sleep and overall health.

Effect modification by adverse CFs was also apparent for life transitions and sleep health in an unexpected direction. Among those with favorable CFs, we observed that high life transition stress was associated with higher prevalence of short sleep duration as well as total sleep score, while these same associations were not apparent among those with adverse CFs. Prior research suggests that life transitions are correlated with psychological distress.^57^ In particular, research has documented how difficulties with a spouse/partner or a romantic relationship break-up, particularly among young adults (18–29 years old), impact mental health.^58^ The age difference in prevalence of life transitions is supported in our data where we observed a higher prevalence of each item in the life transition component (i.e. new romantic relationship the past 12 months, difficulties with former spouse/partner the past 12 month, and started new job the past 12 months) among younger compared to older participants (data not shown). Nonetheless, life transitions may also be viewed positively and as a source of personal growth.^59^ Moreover, new romantic relationships may be viewed positively (e.g. excitement) or as a stressor (e.g. relationship anxiety and uncertainty), while having difficulties with a partner may act primarily as a negative stressor. Starting a new job may be considered positive (e.g. financial resource/stability) or negative/stressful (e.g. challenges related to new demands). Therefore, future research (including mixed methods or qualitative interviews) is warranted to better understand the subjective evaluations or how various life transitions are perceived by BAA women. This approach can inform their influence on associations with sleep health, along with their pathways.

Coping strategies associated with more favorable sleep are also similar to previous findings.^60^ One prior study reported resilience/personal strength components to be associated with lower sleep disturbance among older BAA women with a history of hypertension.^61^ Although we observed few associations between religiosity and sleep health characteristics, religiosity may boost resilience/personal strength through hope for improvement.^62^ While religiosity and spirituality tend to be positive coping strategies among older adults,^62^ the associations may differ in our younger population. In fact, religiosity, while a coping strategy, may also be correlated with stress when BAA women may feel overburdened by community service.^27^

When BAA women experience poor sleep, it is likely due to many life stressors resulting from intersecting racism and sexism that form unique oppressive circumstances (e.g. misogynoir).^63^ For example, psychosocial stressors, such as employment discrimination, activate both the sympathetic nervous system and the HPA axis, which can delay sleep onset, disrupt sleep maintenance, and lead to reduced sleep.^13^ Psychosocial stressors may also impact sleep indirectly through behaviors, such as alcohol consumption and smoking.^64^ Given that adjustment for these factors did not greatly attenuate our observed associations (data not shown), they may not be strong confounders or mediators in our sample. On the other hand, coping strategies, such as resiliency/personal strength, may deactivate these systems and, thus, protect against poor sleep.^28^^,^^29^ Coping strategies, nevertheless, may only serve as temporary methods to mitigate burdens of stress. Stressors due to persistent discrimination practices (including exclusion) through economic, social (including medical), and political systems^27^ likely drive health inequities and manifest, for instance, the phenomena known as the superwoman schema and the suppression of emotions such as anxiety that can manifest as irritation and anger.^27^

Study limitations include potential reverse causation, given that this is a cross-sectional study where participants with current poor sleep may report higher levels of psychosocial stressors and lower levels of coping strategies due to poor sleep. We relied on self-reported data for both stressors/coping strategies and sleep, which may introduce measurement error and/or same-source bias. Self-reported sleep measures also have been shown to overestimate sleep duration compared to objective measures, especially among BAA adults.^65^ Nonetheless, previous human and animal studies using objectively measured sleep dimensions report more stress has been associated with poorer sleep health.^66^^,^^67^ Frequent insomnia symptoms were also not based on a validated scale. Also, the sleep score based on the available data in the SELF study does not include certain aspects of sleep health as defined by Buysse, such as timing and efficiency.^68^ While the SELF study does not include a specific sleep measure to capture the dimension of alertness, waking up feeling unrested can be a proxy for sleepiness or non-alertness. The SELF study also did not refer to a specific time frame (e.g. past 24 hours, past week) when assessing “current” sleep, which may lead to a disconnect between the timing of stressors/coping strategies and the reference period of the sleep questions. Additional research using validated instruments and objective measures is warranted. Another limitation includes lack of data on stress appraisal (e.g. stressor led to actual distress or not). Residual confounding is possible as confounders, such as noise, were not measured but have been shown to influence the stressors-sleep relationship.^69^ Finally, our findings may have limited generalizability since our young BAA participants lived in the Detroit, Michigan area—representing one city and state in the country. Nonetheless, findings may apply to Black women living in comparable environmental and social conditions.

This study has noteworthy strengths. Despite the plethora of research on stress and health, BAA women are an understudied population in this research area. Since BAA women disproportionately experience psychosocial stressors and poor sleep health,^31^ our findings provide important and novel contributions to the literature. An additional strength includes the use of multiple sleep dimensions, which can operate through independent biological mechanisms, making them independently important. We also identified several psychosocial stressors, including racism, as well as coping strategies such as resilience/personal strength, that have been under-investigated concurrently in previous studies. Measuring specific understudied stressors, rather than a global stress measure, is also important to identify which stressors/coping strategies to prioritize for intervention. Similarly, considering the moderating role of adverse CFs and shift work is important as most existing studies limit to a narrow range of life domains, whereas we aimed to encompass a “whole life approach”.

More research focusing on potential interventions is needed. Since BAA women with higher income exhibited higher coping strategies, workplace interventions aimed at improving well-being, addressing mental health challenges, and reducing stress could be effective. Financial incentives (e.g. free counseling services offered) and/or opportunities for time spent engaging in religious/spiritual practices may be helpful to build resilience and social/emotional support. Given our observed associations between racism and medical/crime/family problems in relation to poorer sleep health, addressing medical problems (e.g. improving healthcare quality [by addressing race-based medical gaslighting where experiences of doubt and manipulation make someone feel like their lived realities are not valid], access, and utilization) may improve sleep health outcomes among BAA women. We also reported associations between financial strain and poorer sleep health, where reparations, minimum universal salary, increased minimum wage, subsidized childcare, and student loan forgiveness can help alleviate financial strain. BAA women experience financial strain, at least in part, due to intergenerational racism, wealth inequities, and systems of oppression; therefore, reparations are rooted in international law that include components of cessation, restitution, compensation, satisfaction, and rehabilitation.^70^ Previous cross-sectional research has demonstrated that lower perceived social status is related to worse sleep quality for BAA compared to White adults.^71^ Alleviating financial strain can assist in raising social status by increasing the resources that are accessible to individuals. Finally, interventions should be aimed at addressing early life issues (e.g. increased child tax credits, paid family leave, and/or universal prekindergarten) given the cumulative impact of stressors on health throughout life stages.^72^

Future studies should consider the multidimensional nature of stress, including the proximity (e.g. time window between stressor exposure and assessment; geographical distance from stressor; vicariously experienced stressor), frequency, and intensity of stress. The subsequent influence of stressors on internalizing (e.g. anxiety; substance abuse) and externalizing (e.g. aggression) behaviors should also be considered since racial groups may manifest psychological distress differently.^73^ There remains a need to investigate accumulation of risk as well as assess associations during critical life periods, including using objective physiological data. For example, the in utero period may be a period of vulnerability when subpar fetal growth due to maternal stress may set the stage for later life stress and obesity, especially in combination with their own life stressors.^74^ Lastly, future studies should identify additional potential buffers and resiliency or personal strength components that may mitigate the impact of psychosocial stressors. This may include neighborhood-level factors (e.g. neighborhood social cohesion),^75^ which can enhance positive coping strategies and support to improve sleep health.

In conclusion, we found psychosocial stressors to be associated with poor sleep. For example, racism was associated with shorter sleep duration; financial strain was associated with frequent insomnia symptoms; and emotional distress was associated with shorter sleep duration, frequently experiencing insomnia symptoms, and waking up feeling unrested. In contrast, coping strategies, including social/emotional support and resilience/personal strength, were associated with attaining the recommended hours of sleep and waking up feeling rested. Adverse CFs and shift work modified these associations. Therefore, addressing the identified sources of psychosocial stress may improve sleep health and health sequelae among young BAA women.

## Supplementary Material

Supplementary_Files_zsaf190
