# Supplementary material for: Multiple psychosocial stressors and coping strategies in relation to sleep health
Source: Sleep. 2025 Jul 11;49(3):zsaf190. doi: 10.1093/sleep/zsaf190 (PMC13017407; doi:10.1093/sleep/zsaf190)
Supplement: Supplementary_Files_zsaf190 [file supplementary_files_zsaf190.docx]

**Multiple Psychosocial Stressors and Coping Strategies in Relation to Sleep Health**

Dana M. Alhasan ^1,2^

Frankie LaPorte ^3^

Symielle A. Gaston ^1^

Quaker E. Harmon ^1^

Anissa I. Vines ^4^

John A. McGrath ^3^

W. Braxton Jackson II ^3^

Luciana Giorgio Cosenzo ^5^

Chandra L. Jackson ^1,6^

^1^ Epidemiology Branch, National Institute of Environmental Health Sciences, National Institutes of Health, Department of Health and Human Services, Research Triangle Park, NC, USA

^2^ Department of Epidemiology and Community Health, College of Health and Human Services, University of North Carolina at Charlotte, Charlotte, NC, USA

^3^ DLH, LLC, Bethesda, MD, USA

^4^ Department of Epidemiology, University of North Carolina at Chapel Hill, Chapel Hill, NC, USA

^5^ School of Social Work, University of Alabama, Tuscaloosa, AL, USA

^6^ Intramural Program, National Institute on Minority Health and Health Disparities, National Institutes of Health, Department of Health and Human Services, Bethesda, MD, USA

Please direct correspondence to Dr. Chandra L. Jackson at 111 TW Alexander Drive, MD A3-05, Research Triangle Park, N.C. 27709; telephone: 984-287-3701; fax: 301-480-3290; email: [Chandra.Jackson@nih.gov](mailto:Chandra.Jackson@nih.gov).

| **Supplemental Table 1. Baseline Characteristics of the SELF Participants** | | | | | |
| --- | --- | --- | --- | --- | --- |
| **Demographics** | | **Overall (N=1,693)** | **Study Sample (N=1,678)** | **Excluded from Study (N=15)** |  |
| Age, years | Median, (IQR) | 29.3  (26.3-32.0) | 29.3  (26.3-32.1) | 27.1  (24.6-31.5) |  |
| Annual Household Income | <$20,000 | 766 (45.6%) | 757 (45.4%) | 9 (64.3%) |  |
|  | $20,000-50,000 | 628 (37.4%) | 626 (37.6%) | 2 (14.3%) |  |
|  | >$50,000 | 287 (17.1%) | 284 (17.0%) | 3 (21.4%) |  |
| Education Attainment | High School/GED or Less | 369 (21.8%) | 359 (21.4%) | 10 (71.4%) |  |
|  | Some College/Associates/Techni | 848 (50.1%) | 847 (50.5%) | 1 (7.1%) |  |
|  | Bachelors/Masters/PhD | 475 (28.1%) | 472 (28.1%) | 3 (21.4%) |  |
| Employment Status | Not Employed | 643 (38.0%) | 635 (37.8%) | 8 (53.3%) |  |
|  | < 30 hours | 210 (12.4%) | 209 (12.5%) | 1 (6.7%) |  |
|  | 30+ hours | 840 (49.6%) | 834 (49.7%) | 6 (40.0%) |  |
| Works Rotating Shifts | Yes | 292 (17.2%) | 289 (17.2%) | 3 (20.0%) |  |
|  | No | 1,401 (82.8%) | 1,389 (82.8%) | 12 (80.0%) |  |
| Marital Status | Currently married or living as married | 465 (27.5%) | 463 (27.6%) | 2 (13.3%) |  |
|  | Formerly married or living as married | 236 (13.9%) | 236 (14.1%) | 0 (0.0%) |  |
|  | Single | 992 (58.6%) | 979 (58.3%) | 13 (86.7%) |  |
| Smoking Status | Never | 1,245 (73.5%) | 1,233 (73.5%) | 12 (80.0%) |  |
|  | Former | 125 (7.4%) | 124 (7.4%) | 1 (6.7%) |  |
|  | Current | 323 (19.1%) | 321 (19.1%) | 2 (13.3%) |  |
| Alcohol Consumption | None | 449 (26.5%) | 438 (26.1%) | 11 (73.3%) |  |
|  | Moderate | 553 (32.7%) | 551 (32.8%) | 2 (13.3%) |  |
|  | Heavy | 691 (40.8%) | 689 (41.1%) | 2 (13.3%) |  |
| Physical Activity | Low | 267 (15.8%) | 264 (15.8%) | 3 (20.0%) |  |
|  | Low-to-moderate | 389 (23.0%) | 382 (22.8%) | 7 (46.7%) |  |
|  | Moderate | 390 (23.1%) | 389 (23.3%) | 1 (6.7%) |  |
|  | High | 302 (17.9%) | 299 (17.9%) | 3 (20.0%) |  |
|  | Very high | 340 (20.1%) | 339 (20.3%) | 1 (6.7%) |  |
| Mental Health Diagnosis | Yes | 328 (19.6%) | 325 (19.6%) | 3 (21.4%) |  |
|  | No | 1,347 (80.4%) | 1,336 (80.4%) | 11 (78.6%) |  |
| Body Mass Index | <25 kg/m^2^ | 335 (19.8%) | 333 (19.8%) | 2 (13.3%) |  |
|  | 25-29 kg/m^2^ | 350 (20.7%) | 349 (20.8%) | 1 (6.7%) |  |
|  | 30+ kg/m^2^ | 1,008 (59.5%) | 996 (59.4%) | 12 (80.0%) |  |
| Cardiovascular Risk | Lower risk | 309 (18.3%) | 307 (18.4%) | 2 (13.3%) |  |
|  | Higher risk | 1,376 (81.7%) | 1,363 (81.6%) | 13 (86.7%) |  |
| Asthma | Yes | 331 (19.7%) | 327 (19.7%) | 4 (26.7%) |  |
|  | No | 1,345 (80.3%) | 1,334 (80.3%) | 11 (73.3%) |  |
| Childhood Sleep: Age 5 in bed by 8 | Rarely or never | 154 (9.1%) | 153 (9.1%) | 1 (6.7%) |  |
|  | Sometimes | 377 (22.3%) | 372 (22.2%) | 5 (33.3%) |  |
|  | Often | 403 (23.8%) | 400 (23.9%) | 3 (20.0%) |  |
|  | Most of the time or always | 758 (44.8%) | 752 (44.8%) | 6 (40.0%) |  |
| Childhood Sleep: In a quiet room | Rarely or never | 81 (4.8%) | 79 (4.7%) | 2 (13.3%) |  |
|  | Sometimes | 213 (12.6%) | 209 (12.5%) | 4 (26.7%) |  |
|  | Often | 382 (22.6%) | 379 (22.6%) | 3 (20.0%) |  |
|  | Most of the time or always | 1,017 (60.1%) | 1,011 (60.3%) | 6 (40.0%) |  |
| Childhood Sleep: Unlit or night light | Rarely or never | 188 (11.1%) | 183 (10.9%) | 5 (33.3%) |  |
|  | Sometimes | 244 (14.4%) | 240 (14.3%) | 4 (26.7%) |  |
|  | Often | 321 (19.0%) | 320 (19.1%) | 1 (6.7%) |  |
|  | Most of the time or always | 940 (55.5%) | 935 (55.7%) | 5 (33.3%) |  |
| Childhood Light Sleeper | Yes | 565 (33.4%) | 557 (33.2%) | 8 (53.3%) |  |
|  | No | 1,127 (66.6%) | 1,120 (66.8%) | 7 (46.7%) |  |
| Childhood Sleep Composite | Poor sleep | 791 (46.7%) | 779 (46.4%) | 12 (80.0%) |  |
|  | Good sleep | 902 (53.3%) | 899 (53.6%) | 3 (20.0%) |  |
| Neighborhood Safety at Age 5 | Very unsafe | 96 (5.7%) | 92 (5.5%) | 4 (26.7%) |  |
|  | Somewhat unsafe | 250 (14.8%) | 248 (14.8%) | 2 (13.3%) |  |
|  | Somewhat safe | 696 (41.1%) | 691 (41.2%) | 5 (33.3%) |  |
|  | Very safe | 651 (38.5%) | 647 (38.6%) | 4 (26.7%) |  |
| Neighborhood Safety at Age 10 | Very unsafe | 91 (5.4%) | 89 (5.3%) | 2 (13.3%) |  |
|  | Somewhat unsafe | 281 (16.6%) | 279 (16.6%) | 2 (13.3%) |  |
|  | Somewhat safe | 744 (43.9%) | 738 (44.0%) | 6 (40.0%) |  |
|  | Very safe | 577 (34.1%) | 572 (34.1%) | 5 (33.3%) |  |
| Neighborhood Safety at Age 15 | Very unsafe | 139 (8.2%) | 137 (8.2%) | 2 (13.3%) |  |
|  | Somewhat unsafe | 389 (23.0%) | 387 (23.1%) | 2 (13.3%) |  |
|  | Somewhat safe | 714 (42.2%) | 707 (42.1%) | 7 (46.7%) |  |
|  | Very safe | 451 (26.6%) | 447 (26.6%) | 4 (26.7%) |  |
| Neighborhood Safety Composite | Unsafe | 687 (40.6%) | 680 (40.5%) | 7 (46.7%) |  |
|  | Safe | 1,006 (59.4%) | 998 (59.5%) | 8 (53.3%) |  |
| Childhood Income | High Income | 128 (7.6%) | 124 (7.4%) | 4 (26.7%) |  |
|  | Middle Income | 889 (52.5%) | 883 (52.7%) | 6 (40.0%) |  |
|  | Low Income | 595 (35.2%) | 590 (35.2%) | 5 (33.3%) |  |
|  | Poor | 80 (4.7%) | 80 (4.8%) | 0 (0.0%) |  |
| Childhood Food Insecurity | Yes | 217 (12.8%) | 215 (12.8%) | 2 (13.3%) |  |
|  | No | 1,475 (87.2%) | 1,462 (87.2%) | 13 (86.7%) |  |
| Childhood Economic Composite | Inadequate resources | 721 (42.6%) | 715 (42.6%) | 6 (40.0%) |  |
|  | Adequate resources | 971 (57.4%) | 962 (57.4%) | 9 (60.0%) |  |
| Sleep Duration | Very Short (<6) | 450 (26.6%) | 446 (26.6%) | 4 (26.7%) |  |
|  | Short (6- <7) | 539 (31.8%) | 537 (32.0%) | 2 (13.3%) |  |
|  | Recommended (7-9) | 685 (40.5%) | 677 (40.3%) | 8 (53.3%) |  |
|  | Long (10+) | 19 (1.1%) | 18 (1.1%) | 1 (6.7%) |  |
| Wake Not Rested | Yes | 1,050 (62.0%) | 1,042 (62.1%) | 8 (53.3%) |  |
|  | No | 643 (38.0%) | 636 (37.9%) | 7 (46.7%) |  |
| Insomnia symptoms | Yes | 176 (10.4%) | 175 (10.4%) | 1 (6.7%) |  |
|  | No | 1,517 (89.6%) | 1,503 (89.6%) | 14 (93.3%) |  |
| Sleep Score | 0 (Ideal) | 96 (5.7%) | 94 (5.6%) | 2 (13.3%) |  |
|  | 1 | 618 (36.5%) | 612 (36.5%) | 6 (40.0%) |  |
|  | 2 | 748 (44.2%) | 744 (44.3%) | 4 (26.7%) |  |
|  | 3 (Poor) | 231 (13.6%) | 228 (13.6%) | 3 (20.0%) |  |

We excluded participants with missing data on psychosocial stressors and coping strategies (n=15) resulting in a final analytic sample of 1,678 women

**Supplemental Table 2. Question Items for Components**

| **Components** | **Likert Scale** |
| --- | --- |
| **Emotional Distress** |  |
| How often do you feel the need to suppress or swallow strong feelings of anger | 1 = never,  2 = almost never,  3 = sometimes,  4 = fairly often,  5 = very often |
| How often do you find yourself several days later mentally replaying conversations or events that didn't go your way | 1 = never,  2 = almost never,  3 = sometimes,  4 = fairly often,  5 = very often |
| How often during the past 30 days have you felt that you were unable to control important things in life | 1 = never,  2 = almost never,  3 = sometimes,  4 = fairly often,  5 = very often |
| How often do you yell or shout at yourself or someone else to off steam | 1 = never,  2 = almost never,  3 = sometimes,  4 = fairly often,  5 = very often |
| How often when you are hurt, do you just keep hurt feelings to yourself? | 1 = never,  2 = almost never,  3 = sometimes,  4 = fairly often,  5 = very often |
| How often during the past 30 days have you felt difficulties were piling up so high that you could not overcome them? | 1 = never,  2 = almost never,  3 = sometimes,  4 = fairly often,  5 = very often |
| How stressful is your day-to-day life? | 1= very stressful,  2 = moderately stressful,  3 = mildly stressful,  4= not at all stressful |
| In the past 12 months, have you had a difficult relationship with your current significant other or spouse? | 1 = Yes  2 = No |
| **Experienced Racism** |  |
| In your 20s, how often did you experience racism? | 1 = never,  2 = rarely,  3 = some of the time,  4 = most of the time |
| Before the age of 20, how often did you experience racism? | 1 = never,  2 = rarely,  3 = some of the time,  4 = most of the time |
| In the past 12 months have you experienced racial insults or other acts of discrimination? | 1 = yes  2 = no |
| In the past 5 years how often have you, personally, been called insulting names related to your race by Whites? | 1 = never,  2 = rarely,  3 = occasionally,  4 = frequently |
| How often do you think about your own race? | 1 = never,  2 = rarely, such as once a year,  3 = several times a month,  4 = once a day,  5 = several times a day,  6 = nearly constantly |
| **Perceived Racism** |  |
| When Black people shop, they are followed or watched by security guards or some clerks more often than White shoppers | 1 = strongly disagree,  2 = disagree,  3 = neither agree nor disagree,  4 = agree,  5 = strongly agree |
| In the past 5 years how often have you, personally, been followed or watched by security guards or clerks because of your race? | 1 = never,  2 = rarely,  3 = occasionally,  4 = frequently |
| Black people are still sometimes called insulting names related to their race by Whites | 1 = never,  2 = rarely,  3 = occasionally,  4 = frequently |
| **Financial Strain** |  |
| In the past 12 months have you had difficulty keeping a place to live? | 1 = yes  2 = no |
| In the past 12 months have you had a time when you were not able to afford food or clothing? | 1 = yes  2 = no |
| In the past 12 months have you gotten behind on paying bills? | 1 = yes  2 = no |
| In the past 12 months have you lost a job or have you constantly been afraid of losing a job? | 1 = yes  2 = no |
| In the past 12 months have you moved to a new residence? | 1 = yes  2 = no |
| How difficult is it for you to pay for basic expenses like food, clothing, shelter, medical care, and transportation? | 1 = very difficult to pay expenses,  2 = moderately difficult,  3 = slightly or occasionally difficult,  4 = not at all difficult |
| **Medical/Crime/Family Problems** |  |
| In the past 12 months have you had a major medical problem? | 1 = yes  2 = no |
| In the past 12 months have you had a major problem with your children? | 1 = yes  2 = no |
| In the past 12 months have you had someone very close to you have a major medical problem? | 1 = yes  2 = no |
| In the past 12 months have you experienced a crime where you were threatened with violence or experienced violence? | 1 = yes  2 = no |
| In the past 12 months have you had difficult relationships with your other family members? | 1 = yes  2 = no |
| In the past 12 months have you experienced any other major change or event in your life? | 1 = yes  2 = no |
| In the past 12 months have you experienced a crime other than a violent crime? | 1 = yes  2 = no |
| **Life Transitions** |  |
| In the past 12 months have you had a new romantic relationship? | 1 = yes  2 = no |
| In the past 12 months have you had a difficult relationship with a former spouse or partner? | 1 = yes  2 = no |
| In the past 12 months have you started a new job? | 1 = yes  2 = no |
| **Resilience/Personal Strength** |  |
| How often during the past 30 days have you felt confident about your ability to handle personal problems? | 1 = never,  2 = almost never,  3 = sometimes,  4 = fairly often,  5 = very often |
| How often during the past 30 days have you felt that things were going your way? | 1 = never,  2 = almost never,  3 = sometimes,  4 = fairly often,  5 = very often |
| How often do you talk over a problem and find a fair compromise? | 1 = never,  2 = almost never,  3 = sometimes,  4 = fairly often,  5 = very often |
| **Social/Emotional Support** |  |
| When I was a child, there was someone in my immediate family who believed in me and wanted me to succeed. | 1 = none of the time,  2 = a little of the time,  3 = some of the time,  4 = most of the time,  5 = all of the time |
| When I was a child, there was someone in my immediate family who made me feel important or special. | 1 = none of the time,  2 = a little of the time,  3 = some of the time,  4 = most of the time,  5 = all of the time |
| I can count on someone if I need help, for example, to take me to the doctor or help with daily chores when I am sick. | 1 = none of the time,  2 = a little of the time,  3 = some of the time,  4 = most of the time,  5 = all of the time |
| I can count on someone to provide me with emotional support, someone to confide in about myself or a problem, or who will listen to me when I need to talk. | 1 = none of the time,  2 = a little of the time,  3 = some of the time,  4 = most of the time,  5 = all of the time |
| In general, how many relatives or friends do you feel close to; that is, people you feel at ease with, can talk to about private matters, or call on for help? | 1 = none,  2 = one to two,  3 = three to five,  4 = six to nine,  5 = ten or more |
| **Religiosity** |  |
| How much is religion or spirituality a source of strength and comfort to you? | 1 = not at all,  2 = somewhat,  3 = quite a bit,  4 = very much |
| How important is your religious faith or spirituality to you? | 1 = not at all,  2 = somewhat important,  3 = moderately important,  4 = very important, |
| How often do you pray or meditate? | 1 = never,  2 = less than once a month,  3 = monthly or a few days per month,  4 = weekly or a few days per week,  5 = every day,  6 = a few times per day,  7 = several times per day |

**Supplemental Table 3. Rotated Component Loadings**

| **Components** | **Loading** | **Time Range of Questions** |
| --- | --- | --- |
| **Emotional Distress (30.8% Variance)** |  |  |
| Suppress/swallow strong feelings of anger | 0.767 | General frequency |
| Replaying conversations or events that didn't go your way | 0.738 | General frequency |
| Unable to control important things in life | 0.666 | Past 30 days |
| Yelling/Shouting to let off steam | 0.661 | General frequency |
| Keeping hurt feelings to yourself | 0.636 | General frequency |
| Could not overcome difficulties | 0.624 | Past 30 days |
| Day-to-day stress level | 0.388 | Past 12 months |
| Difficulties with current significant other or spouse | 0.395 | Past 12 months |
| **Experienced Racism (10.8% Variance)** |  |  |
| Experienced racism in 20s | 0.859 | General frequency |
| Experienced racism before age 20 | 0.745 | General frequency |
| Experienced racism in last year | 0.696 | Past 12 months |
| Experienced slurs from Whites | 0.684 | Past 5 years |
| Thought about own race | 0.626 | General frequency |
| **Perceived Racism (5.2% Variance)** |  |  |
| Blacks watched more closely than whites while shopping | 0.638 | General frequency |
| Felt being watched due to race | 0.542 | Past 5 years |
| Black still subject to slurs from whites | 0.477 | General frequency |
| **Financial Strain (9.2% Variance)** |  |  |
| Difficulty keeping home | 0.748 | Past 12 months |
| Couldn't afford food/clothes | 0.676 | Past 12 months |
| Fell behind in bills | 0.639 | Past 12 months |
| Lost job or feared loss of job | 0.497 | Past 12 months |
| Moved to new home | 0.447 | Past 12 months |
| Difficulty paying basic expenses | -0.740 | Past 12 months |
| **Medical/Crime/Family Problems (8.1% Variance)** |  |  |
| Major medical problem | 0.659 | Past 12 months |
| Major problem with kids | 0.565 | Past 12 months |
| Someone close had major medical problem | 0.524 | Past 12 months |
| Victim of violent crime | 0.485 | Past 12 months |
| Problems with other family | 0.485 | Past 12 months |
| Other major change/event | 0.453 | Past 12 months |
| Victim of non-violent crime | 0.320 | Past 12 months |
| **Life Transitions (5.3% Variance)** |  |  |
| New romantic relationship | 0.683 | Past 12 months |
| Difficulties with former spouse/partner | 0.638 | Past 12 months |
| Started new job | 0.452 | Past 12 months |
| **Resilience/Personal Strength (5.8% Variance)** |  |  |
| Confident about ability to handle personal problems | 0.816 | Past 30 days |
| Feeling things going your way | 0.787 | Past 30 days |
| Talking over a problem and finding a compromise | 0.479 | General frequency |
| **Social/Emotional Support (18.1% Variance)** |  |  |
| Family believed in/supported as a child | 0.887 | General frequency |
| Family made feel special as a child | 0.848 | General frequency |
| Can count on someone for help | 0.731 | General frequency |
| Can count on someone for emotional support | 0.722 | General frequency |
| Number of people feel close to | 0.666 | General frequency |
| **Religiosity (6.6% Variance)** |  |  |
| Religion/spirituality is strength/comfort | 0.918 | General frequency |
| Importance of faith | 0.914 | General frequency |
| Frequency of prayer/meditation | 0.824 | General frequency |

* Components in the unrotated solution accounted for 57% total variance. The table reports each component’s share of rotated variance, which sums to 100%.

Component loadings greater than or equal to 0.30 are shown.

# **Supplemental Table 4. Sociodemographic Characteristics of SELF Participants by Psychosocial Stressors and Coping Strategies, Study of Environment, Lifestyle, and Fibroids, 2010-2012 (N=1,661)**

|  | | **Emotional Distress** | | **Experienced Racism** | | **Financial Strain** | | **Medical/Crime/Family Problems** | | **Perceived Racism** | | **Religiosity** | | **Resilience/Personal Strength** | | **Social/Emotional Support** | | **Transition** | |
| --- | --- | --- | --- | --- | --- | --- | --- | --- | --- | --- | --- | --- | --- | --- | --- | --- | --- | --- | --- |
|  | | **Low** | **High** | **Low** | **High** | **Low** | **High** | **Low** | **High** | **Low** | **High** | **Low** | **High** | **Low** | **High** | **Low** | **High** | **Low** | **High** |
| Age | 23 - 29 | 469 (55.9%) | 469 (55.9%) | 458 (54.6%) | 480 (57.2%) | 457 (54.5%) | 481 (57.3%) | **515 (61.4%)** | **423 (50.4%)** | **491 (58.5%)** | **447 (53.3%)** | **491 (58.5%)** | **447 (53.3%)** | 476 (56.7%) | 462 (55.1%) | 453 (54.0%) | 485 (57.8%) | **419 (49.9%)** | **519 (61.9%)** |
|  | 30 - 35 | 370 (44.1%) | 370 (44.1%) | 381 (45.4%) | 359 (42.8%) | 382 (45.5%) | 358 (42.7%) | **324 (38.6%)** | **416 (49.6%)** | **348 (41.5%)** | **392 (46.7%)** | **348 (41.5%)** | **392 (46.7%)** | 363 (43.3%) | 377 (44.9%) | 386 (46.0%) | 354 (42.2%) | **420 (50.1%)** | **320 (38.1%)** |
| Family Income | <20k | 372 (44.7%) | 385 (46.2%) | **431 (51.7%)** | **326 (39.1%)** | **288 (34.5%)** | **469 (56.3%)** | **371 (44.5%)** | **386 (46.3%)** | 380 (45.5%) | 377 (45.3%) | 392 (47.0%) | 365 (43.8%) | **420 (50.2%)** | **337 (40.6%)** | **455 (54.6%)** | **302 (36.3%)** | **361 (43.2%)** | **396 (47.6%)** |
|  | 20-50k | 315 (37.8%) | 311 (37.3%) | **307 (36.9%)** | **319 (38.2%)** | **348 (41.7%)** | **278 (33.4%)** | **302 (36.2%)** | **324 (38.9%)** | 313 (37.5%) | 313 (37.6%) | 311 (37.3%) | 315 (37.8%) | **284 (34.0%)** | **342 (41.2%)** | **279 (33.5%)** | **347 (41.7%)** | **304 (36.4%)** | **322 (38.7%)** |
|  | 50K+ | 146 (17.5%) | 138 (16.5%) | **95 (11.4%)** | **189 (22.7%)** | **198 (23.7%)** | **86 (10.3%)** | **161 (19.3%)** | **123 (14.8%)** | 142 (17.0%) | 142 (17.1%) | 131 (15.7%) | 153 (18.4%) | **132 (15.8%)** | **152 (18.3%)** | **100 (12.0%)** | **184 (22.1%)** | **170 (20.4%)** | **114 (13.7%)** |
| Education | High School/GED or Less | 194 (23.1%) | 165 (19.7%) | **244 (29.1%)** | **115 (13.7%)** | **158 (18.8%)** | **201 (24.0%)** | **189 (22.5%)** | **170 (20.3%)** | 191 (22.8%) | 168 (20.0%) | **211 (25.1%)** | **148 (17.6%)** | **200 (23.8%)** | **159 (19.0%)** | **243 (29.0%)** | **116 (13.8%)** | 179 (21.3%) | 180 (21.5%) |
|  | Some College/Associates/Technical | 401 (47.8%) | 446 (53.2%) | **426 (50.8%)** | **421 (50.2%)** | **380 (45.3%)** | **467 (55.7%)** | **378 (45.1%)** | **469 (55.9%)** | 426 (50.8%) | 421 (50.2%) | **416 (49.6%)** | **431 (51.4%)** | **417 (49.7%)** | **430 (51.3%)** | **425 (50.7%)** | **422 (50.3%)** | 421 (50.2%) | 426 (50.8%) |
|  | Bachelors/Masters/PhD | 244 (29.1%) | 228 (27.2%) | **169 (20.1%)** | **303 (36.1%)** | **301 (35.9%)** | **171 (20.4%)** | **272 (32.4%)** | **200 (23.8%)** | 222 (26.5%) | 250 (29.8%) | **212 (25.3%)** | **260 (31.0%)** | **222 (26.5%)** | **250 (29.8%)** | **171 (20.4%)** | **301 (35.9%)** | 239 (28.5%) | 233 (27.8%) |
| Employment | Not Employed | 318 (37.9%) | 317 (37.8%) | **374 (44.6%)** | **261 (31.1%)** | **258 (30.8%)** | **377 (44.9%)** | **290 (34.6%)** | **345 (41.1%)** | 307 (36.6%) | 328 (39.1%) | 328 (39.1%) | 307 (36.6%) | **345 (41.1%)** | **290 (34.6%)** | **356 (42.4%)** | **279 (33.3%)** | **363 (43.3%)** | **272 (32.4%)** |
|  | < 30 hours | 107 (12.8%) | 102 (12.2%) | **88 (10.5%)** | **121 (14.4%)** | **96 (11.4%)** | **113 (13.5%)** | **105 (12.5%)** | **104 (12.4%)** | 112 (13.3%) | 97 (11.6%) | 94 (11.2%) | 115 (13.7%) | **88 (10.5%)** | **121 (14.4%)** | **117 (13.9%)** | **92 (11.0%)** | **76 (9.1%)** | **133 (15.9%)** |
|  | 30+ hours | 414 (49.3%) | 420 (50.1%) | **377 (44.9%)** | **457 (54.5%)** | **485 (57.8%)** | **349 (41.6%)** | **444 (52.9%)** | **390 (46.5%)** | 420 (50.1%) | 414 (49.3%) | 417 (49.7%) | 417 (49.7%) | **406 (48.4%)** | **428 (51.0%)** | **366 (43.6%)** | **468 (55.8%)** | **400 (47.7%)** | **434 (51.7%)** |
| Works rotating shifts | Yes | 142 (16.9%) | 147 (17.5%) | 131 (15.6%) | 158 (18.8%) | 135 (16.1%) | 154 (18.4%) | 155 (18.5%) | 134 (16.0%) | **161 (19.2%)** | **128 (15.3%)** | 144 (17.2%) | 145 (17.3%) | 145 (17.3%) | 144 (17.2%) | 147 (17.5%) | 142 (16.9%) | **121 (14.4%)** | **168 (20.0%)** |
|  | No | 697 (83.1%) | 692 (82.5%) | 708 (84.4%) | 681 (81.2%) | 704 (83.9%) | 685 (81.6%) | 684 (81.5%) | 705 (84.0%) | **678 (80.8%)** | **711 (84.7%)** | 695 (82.8%) | 694 (82.7%) | 694 (82.7%) | 695 (82.8%) | 692 (82.5%) | 697 (83.1%) | **718 (85.6%)** | **671 (80.0%)** |
| Marital Status | Currently married or living as married | **204 (24.3%)** | **259 (30.9%)** | **195 (23.2%)** | **268 (31.9%)** | 232 (27.7%) | 231 (27.5%) | **203 (24.2%)** | **260 (31.0%)** | **255 (30.4%)** | **208 (24.8%)** | **218 (26.0%)** | **245 (29.2%)** | 230 (27.4%) | 233 (27.8%) | 214 (25.5%) | 249 (29.7%) | **316 (37.7%)** | **147 (17.5%)** |
|  | Formerly married or living as married | **119 (14.2%)** | **117 (13.9%)** | **117 (13.9%)** | **119 (14.2%)** | 116 (13.8%) | 120 (14.3%) | **99 (11.8%)** | **137 (16.3%)** | **109 (13.0%)** | **127 (15.1%)** | **104 (12.4%)** | **132 (15.7%)** | 114 (13.6%) | 122 (14.5%) | 125 (14.9%) | 111 (13.2%) | **88 (10.5%)** | **148 (17.6%)** |
|  | Single | **516 (61.5%)** | **463 (55.2%)** | **527 (62.8%)** | **452 (53.9%)** | 491 (58.5%) | 488 (58.2%) | **537 (64.0%)** | **442 (52.7%)** | **475 (56.6%)** | **504 (60.1%)** | **517 (61.6%)** | **462 (55.1%)** | 495 (59.0%) | 484 (57.7%) | 500 (59.6%) | 479 (57.1%) | **435 (51.8%)** | **544 (64.8%)** |
| Smoking Status | Never | 626 (74.6%) | 607 (72.3%) | 599 (71.4%) | 634 (75.6%) | **674 (80.3%)** | **559 (66.6%)** | **640 (76.3%)** | **593 (70.7%)** | 621 (74.0%) | 612 (72.9%) | **592 (70.6%)** | **641 (76.4%)** | 610 (72.7%) | 623 (74.3%) | **558 (66.5%)** | **675 (80.5%)** | 627 (74.7%) | 606 (72.2%) |
|  | Former | 61 (7.3%) | 63 (7.5%) | 65 (7.7%) | 59 (7.0%) | **63 (7.5%)** | **61 (7.3%)** | **43 (5.1%)** | **81 (9.7%)** | 55 (6.6%) | 69 (8.2%) | **60 (7.2%)** | **64 (7.6%)** | 60 (7.2%) | 64 (7.6%) | **74 (8.8%)** | **50 (6.0%)** | 68 (8.1%) | 56 (6.7%) |
|  | Current | 152 (18.1%) | 169 (20.1%) | 175 (20.9%) | 146 (17.4%) | **102 (12.2%)** | **219 (26.1%)** | **156 (18.6%)** | **165 (19.7%)** | 163 (19.4%) | 158 (18.8%) | **187 (22.3%)** | **134 (16.0%)** | 169 (20.1%) | 152 (18.1%) | **207 (24.7%)** | **114 (13.6%)** | 144 (17.2%) | 177 (21.1%) |
| Alcohol Consumption | None | **243 (29.0%)** | **195 (23.2%)** | **252 (30.0%)** | **186 (22.2%)** | **235 (28.0%)** | **203 (24.2%)** | 232 (27.7%) | 206 (24.6%) | 222 (26.5%) | 216 (25.7%) | **180 (21.5%)** | **258 (30.8%)** | 226 (26.9%) | 212 (25.3%) | 237 (28.2%) | 201 (24.0%) | **257 (30.6%)** | **181 (21.6%)** |
|  | Moderate | **275 (32.8%)** | **276 (32.9%)** | **243 (29.0%)** | **308 (36.7%)** | **300 (35.8%)** | **251 (29.9%)** | 284 (33.8%) | 267 (31.8%) | 278 (33.1%) | 273 (32.5%) | **268 (31.9%)** | **283 (33.7%)** | 256 (30.5%) | 295 (35.2%) | 266 (31.7%) | 285 (34.0%) | **263 (31.3%)** | **288 (34.3%)** |
|  | Heavy | **321 (38.3%)** | **368 (43.9%)** | **344 (41.0%)** | **345 (41.1%)** | **304 (36.2%)** | **385 (45.9%)** | 323 (38.5%) | 366 (43.6%) | 339 (40.4%) | 350 (41.7%) | **391 (46.6%)** | **298 (35.5%)** | 357 (42.6%) | 332 (39.6%) | 336 (40.0%) | 353 (42.1%) | **319 (38.0%)** | **370 (44.1%)** |
| Physical Activity | Low | 124 (14.9%) | 140 (16.7%) | **142 (17.0%)** | **122 (14.6%)** | **132 (15.8%)** | **132 (15.8%)** | 134 (16.0%) | 130 (15.5%) | 127 (15.2%) | 137 (16.4%) | 134 (16.0%) | 130 (15.5%) | 135 (16.2%) | 129 (15.4%) | 120 (14.4%) | 144 (17.2%) | 137 (16.4%) | 127 (15.2%) |
|  | Low-to-moderate | 187 (22.4%) | 195 (23.3%) | **197 (23.6%)** | **185 (22.1%)** | **216 (25.8%)** | **166 (19.9%)** | 194 (23.2%) | 188 (22.4%) | 180 (21.5%) | 202 (24.2%) | 182 (21.8%) | 200 (23.9%) | 186 (22.3%) | 196 (23.4%) | 200 (23.9%) | 182 (21.7%) | 195 (23.3%) | 187 (22.3%) |
|  | Moderate | 216 (25.9%) | 173 (20.6%) | **209 (25.0%)** | **180 (21.5%)** | **198 (23.7%)** | **191 (22.8%)** | 194 (23.2%) | 195 (23.3%) | 208 (24.9%) | 181 (21.7%) | 203 (24.3%) | 186 (22.2%) | 207 (24.8%) | 182 (21.7%) | 199 (23.8%) | 190 (22.7%) | 196 (23.4%) | 193 (23.1%) |
|  | High | 138 (16.5%) | 161 (19.2%) | **139 (16.6%)** | **160 (19.1%)** | **149 (17.8%)** | **150 (17.9%)** | 147 (17.6%) | 152 (18.1%) | 149 (17.8%) | 150 (17.9%) | 153 (18.3%) | 146 (17.4%) | 147 (17.6%) | 152 (18.1%) | 141 (16.9%) | 158 (18.9%) | 153 (18.3%) | 146 (17.4%) |
|  | Very high | 170 (20.4%) | 169 (20.2%) | **149 (17.8%)** | **190 (22.7%)** | **142 (17.0%)** | **197 (23.6%)** | 166 (19.9%) | 173 (20.6%) | 173 (20.7%) | 166 (19.9%) | 164 (19.6%) | 175 (20.9%) | 160 (19.2%) | 179 (21.4%) | 176 (21.1%) | 163 (19.5%) | 155 (18.5%) | 184 (22.0%) |
| Mental Illness Diagnosis | Yes | **124 (14.9%)** | **201 (24.3%)** | 151 (18.2%) | 174 (20.9%) | **137 (16.5%)** | **188 (22.7%)** | **121 (14.6%)** | **204 (24.5%)** | 160 (19.2%) | 165 (20.0%) | 163 (19.7%) | 162 (19.4%) | **189 (22.8%)** | **136 (16.3%)** | **191 (23.0%)** | **134 (16.1%)** | 167 (20.0%) | 158 (19.1%) |
|  | No | **710 (85.1%)** | **626 (75.7%)** | 678 (81.8%) | 658 (79.1%) | **694 (83.5%)** | **642 (77.3%)** | **709 (85.4%)** | **627 (75.5%)** | 675 (80.8%) | 661 (80.0%) | 665 (80.3%) | 671 (80.6%) | **640 (77.2%)** | **696 (83.7%)** | **639 (77.0%)** | **697 (83.9%)** | 667 (80.0%) | 669 (80.9%) |
| Body Mass Index | <25 | 180 (21.5%) | 153 (18.2%) | 166 (19.8%) | 167 (19.9%) | **188 (22.4%)** | **145 (17.3%)** | 176 (21.0%) | 157 (18.7%) | 180 (21.5%) | 153 (18.2%) | 154 (18.4%) | 179 (21.3%) | 155 (18.5%) | 178 (21.2%) | 157 (18.7%) | 176 (21.0%) | 148 (17.6%) | 185 (22.1%) |
|  | 25-29 | 170 (20.3%) | 179 (21.3%) | 164 (19.5%) | 185 (22.1%) | **177 (21.1%)** | **172 (20.5%)** | 184 (21.9%) | 165 (19.7%) | 162 (19.3%) | 187 (22.3%) | 182 (21.7%) | 167 (19.9%) | 174 (20.7%) | 175 (20.9%) | 168 (20.0%) | 181 (21.6%) | 183 (21.8%) | 166 (19.8%) |
|  | 30+ | 489 (58.3%) | 507 (60.4%) | 509 (60.7%) | 487 (58.0%) | **474 (56.5%)** | **522 (62.2%)** | 479 (57.1%) | 517 (61.6%) | 497 (59.2%) | 499 (59.5%) | 503 (60.0%) | 493 (58.8%) | 510 (60.8%) | 486 (57.9%) | 514 (61.3%) | 482 (57.4%) | 508 (60.5%) | 488 (58.2%) |
| Cardiovascular Risk | Lower risk | 168 (20.1%) | 139 (16.6%) | 157 (18.8%) | 150 (17.9%) | **174 (20.9%)** | **133 (15.9%)** | 165 (19.7%) | 142 (17.0%) | 166 (19.8%) | 141 (16.9%) | 142 (17.0%) | 165 (19.7%) | 145 (17.4%) | 162 (19.4%) | 146 (17.5%) | 161 (19.3%) | **131 (15.7%)** | **176 (21.1%)** |
|  | Higher risk | 667 (79.9%) | 696 (83.4%) | 677 (81.2%) | 686 (82.1%) | **660 (79.1%)** | **703 (84.1%)** | 671 (80.3%) | 692 (83.0%) | 672 (80.2%) | 691 (83.1%) | 692 (83.0%) | 671 (80.3%) | 690 (82.6%) | 673 (80.6%) | 688 (82.5%) | 675 (80.7%) | **706 (84.3%)** | **657 (78.9%)** |
|  |  |  |  |  |  |  |  |  |  |  |  |  |  |  |  |  |  |  |  |
| Bolded values represent significant differences (p < 0.05) based on Chi-Square test. | |  |  |  |  |  |  |  |  |  |  |  |  |  |  |  |  |  |  |

# **Supplemental Table 5. Prevalence Ratios of Sleep Health by Stressors and Coping Strategies Stratified by Childhood Sleep, Study of Environment, Lifestyle, and Fibroids, 2010-2012 (N=1,661)**

| **Sleep Outcomes** | **Very Short Sleep (<6 hours) vs. 7-9 hours** | | **Short Sleep (<7 hours) vs. 7-9 hours** | | **Frequent Insomnia^a^ Symptoms (≥ 15 days per month** | | **Wake up feeling unrested (≥ 4 days per week)** | | **Total Sleep Score^b^** | |
| --- | --- | --- | --- | --- | --- | --- | --- | --- | --- | --- |
|  | **PR (95% CI)** | **PR (95% CI)** | **PR (95% CI)** | **PR (95% CI)** | **PR (95% CI)** | **PR (95% CI)** | **PR (95% CI)** | **PR (95% CI)** | **PR (95% CI)** | **PR (95% CI)** |
| **Childhood Sleep** | **Poor** | **Non-poor** | **Poor** | **Non-poor** | **Poor** | **Non-poor** | **Poor** | **Non-poor** | **Poor** | **Non-poor** |
| Emotional Distress | 1.03  (0.82, 1.29) | 1.32  (1.05, 1.66) | 1.18  (1.05, 1.33) | 1.10  (0.98, 1.23) | 1.82  (1.18, 2.80) | 2.80  (1.76, 4.45) | **1.20**  **(1.08, 1.34)** | **1.50**  **(1.34, 1.68)** | 1.05  (0.98, 1.12) | 0.99  (0.94, 1.06) |
| Experienced Racism | 1.02  (0.82, 1.28) | 1.18  (0.94, 1.49) | 1.10  (0.98, 1.24) | 1.17  (1.04, 1.31) | 0.94  (0.63, 1.41) | 1.56  (1.02, 2.39) | 1.10  (0.99, 1.23) | 1.00  (0.89, 1.11) | 1.06  (0.99, 1.14) | 1.08  (1.02, 1.15) |
| Perceived Racism | **0.74**  **(0.59, 0.93)** | **1.17**  **(0.93, 1.47)** | 1.00  (0.89, 1.12) | 1.00  (0.90, 1.12) | 1.03  (0.69, 1.53) | 1.28  (0.85, 1.91) | 1.02  (0.92, 1.13) | 1.08  (0.98, 1.21) | 1.02  (0.95, 1.08) | 0.98  (0.92, 1.04) |
| Financial Strain | 1.08  (0.86, 1.36) | 1.23  (0.98, 1.56) | 1.02  (0.90, 1.14) | 1.05  (0.93, 1.17) | 1.65  (1.07, 2.54) | 1.23  (0.82, 1.85) | 1.05  (0.94, 1.17) | 1.03  (0.93, 1.15) | 0.97  (0.91, 1.04) | 1.04  (0.98, 1.11) |
| Medical/Crime/Family Problems | 1.09  (0.87, 1.37) | 1.37  (1.08, 1.73) | 1.01  (0.90, 1.14) | 1.04  (0.93, 1.17) | 0.97  (0.65, 1.44) | 1.33  (0.88, 2.02) | 1.11  (0.99, 1.23) | 1.12  (1.01, 1.25) | 1.01  (0.95, 1.08) | 1.00  (0.95, 1.07) |
| Life Transitions | **0.76**  **(0.61, 0.96)** | **1.13**  **(0.90, 1.43)** | 0.87  (0.78, 0.98) | 1.01  (0.90, 1.13) | 0.81  (0.54, 1.22) | 0.91  (0.61, 1.35) | 0.92  (0.82, 1.02) | 0.96  (0.86, 1.07) | 0.96  (0.90, 1.03) | 1.02  (0.96, 1.09) |
| Resilience/Personal Strength | 0.86  (0.68, 1.07) | 0.79  (0.63, 1.00) | 0.88  (0.78, 0.99) | 0.91  (0.82, 1.02) | 0.63  (0.41, 0.97) | 0.96  (0.65, 1.42) | 0.95  (0.85, 1.06) | 0.85  (0.77, 0.95) | 0.97  (0.91, 1.04) | 1.00  (0.94, 1.06) |
| Social/Emotional Support | 0.90  (0.71, 1.14) | 0.74  (0.59, 0.93) | 0.94  (0.83, 1.06) | 0.93  (0.83, 1.04) | 0.93  (0.62, 1.41) | 0.80  (0.54, 1.20) | 0.90  (0.81, 1.01) | 0.85  (0.76, 0.94) | 0.98  (0.91, 1.05) | 1.01  (0.95, 1.07) |
| Religiosity | 1.17  (0.93, 1.45) | 0.92  (0.73, 1.16) | 1.02  (0.91, 1.14) | 1.09  (0.98, 1.22) | 1.10  (0.74, 1.65) | 1.24  (0.84, 1.85) | 0.96  (0.86, 1.07) | 1.00  (0.90, 1.11) | 1.01  (0.94, 1.08) | 1.06  (0.99, 1.12) |

PR=Prevalence Ratio; CI=Confidence Interval

Model adjusted for age (measured continuously), employment status, educational attainment, marital status, annual household income, cardiovascular risk, mental health diagnoses, asthma, smoking status, alcohol consumption and physical activity.

Bolded estimates represent significance at p < 0.05 for the type3 test of the interaction term.

^a^Frequent insomnia symptoms defined as either trouble falling asleep or waking up during the night, 15+ days a month.

^b^Higher total sleep score indicates poorer sleep. Total sleep score was calculated as one point for each of the following: very short, short, or long sleep duration (where very short and short sleep were mutually exclusive); frequent insomnia symptoms; and waking up feeling unrested where very short and short sleep were mutually exclusive. The total poor sleep score ranged from 0-3 with a higher score indicating poorer sleep.

Childhood sleep was measured as poor sleep if participants marked any of the following as “never” or “rarely”: 1) “in bed by 8 pm”, 2) “dark room/ night light” and 3) “quiet room”.

# **Supplemental Table 6. Prevalence Ratios of Sleep Health by Stressors and Coping Strategies Stratified by Childhood Light Sleeper, Study of Environment, Lifestyle, and Fibroids, 2010-2012 (N=1,661)**

| **Sleep Outcomes** | **Very Short Sleep (<6 hours) vs. 7-9 hours** | | **Short Sleep (<7 hours) vs. 7-9 hours** | | **Frequent Insomnia^a^ Symptoms (≥ 15 days per month** | | **Wake up feeling unrested (≥ 4 days per week)** | | **Total Sleep Score^b^** | |
| --- | --- | --- | --- | --- | --- | --- | --- | --- | --- | --- |
|  | **PR (95% CI)** | **PR (95% CI)** | **PR (95% CI)** | **PR (95% CI)** | **PR (95% CI)** | **PR (95% CI)** | **PR (95% CI)** | **PR (95% CI)** | **PR (95% CI)** | **PR (95% CI)** |
| **Childhood Light Sleeper** | **Yes** | **No** | **Yes** | **No** | **Yes** | **No** | **Yes** | **No** | **Yes** | **No** |
| Emotional Distress | 1.02  (0.79, 1.30) | 1.27  (1.03, 1.56) | 1.08  (0.94, 1.24) | 1.17  (1.05, 1.29) | 2.17  (1.28, 3.67) | 2.27  (1.53, 3.36) | 1.30  (1.14, 1.48) | 1.38  (1.25, 1.52) | 1.03  (0.95, 1.11) | 1.02  (0.96, 1.08) |
| Experienced Racism | 1.05  (0.81, 1.35) | 1.13  (0.92, 1.39) | 1.06  (0.93, 1.21) | 1.18  (1.06, 1.31) | 1.63  (1.02, 2.61) | 1.00  (0.70, 1.43) | 1.06  (0.93, 1.20) | 1.04  (0.94, 1.14) | 1.08  (1.00, 1.16) | 1.07  (1.01, 1.13) |
| Perceived Racism | **0.72**  **(0.56, 0.93)** | **1.10**  **(0.90, 1.35)** | 0.94  (0.82, 1.08) | 1.04  (0.94, 1.15) | 1.06  (0.67, 1.66) | 1.25  (0.88, 1.78) | **0.91**  **(0.80, 1.04)** | **1.14**  **(1.04, 1.25)** | 1.01  (0.94, 1.09) | 0.99  (0.94, 1.04) |
| Financial Strain | 1.32  (1.01, 1.73) | 1.06  (0.86, 1.31) | 1.06  (0.92, 1.21) | 1.01  (0.91, 1.13) | 1.31  (0.81, 2.13) | 1.43  (0.98, 2.08) | 1.02  (0.90, 1.17) | 1.05  (0.95, 1.16) | 0.96  (0.89, 1.04) | 1.03  (0.98, 1.09) |
| Medical/Crime/Family Problems | 1.21  (0.94, 1.56) | 1.23  (0.99, 1.52) | 1.13  (0.99, 1.30) | 0.98  (0.88, 1.08) | 1.16  (0.73, 1.85) | 1.10  (0.76, 1.60) | 1.22  (1.07, 1.40) | 1.06  (0.96, 1.17) | 1.00  (0.92, 1.08) | 1.01  (0.96, 1.07) |
| Life Transitions | 0.79  (0.61, 1.01) | 1.01  (0.82, 1.25) | 0.94  (0.82, 1.07) | 0.94  (0.85, 1.05) | 0.65  (0.40, 1.03) | 1.01  (0.70, 1.46) | 0.98  (0.86, 1.12) | 0.92  (0.83, 1.01) | 0.96  (0.89, 1.04) | 1.01  (0.95, 1.07) |
| Resilience/Personal Strength | 0.74  (0.57, 0.95) | 0.87  (0.71, 1.07) | 0.95  (0.83, 1.08) | 0.87  (0.78, 0.96) | 0.90  (0.57, 1.43) | 0.73  (0.50, 1.06) | 0.91  (0.80, 1.04) | 0.89  (0.81, 0.97) | 1.04  (0.96, 1.12) | 0.96  (0.91, 1.01) |
| Social/Emotional Support | 0.68  (0.51, 0.89) | 0.89  (0.73, 1.10) | 0.86  (0.75, 0.99) | 0.97  (0.88, 1.08) | 0.79  (0.49, 1.28) | 0.89  (0.62, 1.29) | 0.80  (0.70, 0.92) | 0.90  (0.82, 0.99) | 1.03  (0.95, 1.11) | 0.98  (0.92, 1.04) |
| Religiosity | 1.15  (0.90, 1.48) | 0.97  (0.79, 1.20) | 1.08  (0.94, 1.23) | 1.05  (0.95, 1.16) | 1.46  (0.92, 2.32) | 1.02  (0.71, 1.47) | 0.99  (0.87, 1.13) | 0.97  (0.88, 1.07) | 1.05  (0.98, 1.14) | 1.02  (0.97, 1.08) |

PR=Prevalence Ratio; CI=Confidence Interval

Model adjusted for age (measured continuously), employment status, educational attainment, marital status, annual household income, cardiovascular risk, mental health diagnoses, asthma, smoking status, alcohol consumption and physical activity.

Bolded estimates represent significance at p < 0.05 for the type3 test of the interaction term.

^a^Frequent insomnia symptoms defined as either trouble falling asleep or waking up during the night, 15+ days a month.

^b^Higher total sleep score indicates poorer sleep. Total sleep score was calculated as one point for each of the following: very short, short, or long sleep duration (where very short and short sleep were mutually exclusive); frequent insomnia symptoms; and waking up feeling unrested where very short and short sleep were mutually exclusive. The total poor sleep score ranged from 0-3 with a higher score indicating poorer sleep.

Participants responded yes/no to “When you were around 5 years old, were you a light sleeper, that is easily awakened?”.

# **Supplemental Table 7. Prevalence Ratios of Sleep Health by Stressors and Coping Strategies Stratified by Childhood Safety, Study of Environment, Lifestyle, and Fibroids, 2010-2012 (N=1,661)**

| **Sleep Outcomes** | **Very Short Sleep (<6 hours) vs. 7-9 hours** | | **Short Sleep (<7 hours) vs. 7-9 hours** | | **Frequent Insomnia^a^ Symptoms (≥ 15 days per month** | | **Wake up feeling unrested (≥ 4 days per week)** | | **Total Sleep Score^b^** | |
| --- | --- | --- | --- | --- | --- | --- | --- | --- | --- | --- |
|  | **PR (95% CI)** | **PR (95% CI)** | **PR (95% CI)** | **PR (95% CI)** | **PR (95% CI)** | **PR (95% CI)** | **PR (95% CI)** | **PR (95% CI)** | **PR (95% CI)** | **PR (95% CI)** |
| **Childhood Safety** | **Safe** | **Unsafe** | **Safe** | **Unsafe** | **Safe** | **Unsafe** | **Safe** | **Unsafe** | **Safe** | **Unsafe** |
| Emotional Distress | 1.23  (0.97, 1.57) | 1.11  (0.89, 1.38) | 1.09  (0.97, 1.24) | 1.17  (1.05, 1.30) | 2.21  (1.38, 3.53) | 2.28  (1.48, 3.52) | 1.25  (1.12, 1.40) | 1.42  (1.27, 1.58) | 1.01  (0.94, 1.08) | 1.03  (0.97, 1.09) |
| Experienced Racism | 1.17  (0.92, 1.48) | 1.02  (0.82, 1.28) | 1.12  (0.99, 1.27) | 1.14  (1.03, 1.27) | 1.18  (0.77, 1.80) | 1.22  (0.83, 1.79) | 0.97  (0.87, 1.09) | 1.08  (0.97, 1.20) | 1.10  (1.02, 1.18) | 1.06  (1.00, 1.12) |
| Perceived Racism | 0.99  (0.79, 1.26) | 0.89  (0.71, 1.10) | 1.00  (0.89, 1.13) | 1.00  (0.90, 1.11) | 1.22  (0.81, 1.85) | 1.08  (0.74, 1.58) | 1.02  (0.92, 1.14) | 1.08  (0.97, 1.19) | 1.02  (0.95, 1.09) | 0.98  (0.92, 1.04) |
| Financial Strain | 1.03  (0.80, 1.32) | 1.23  (0.99, 1.53) | 1.07  (0.94, 1.21) | 1.00  (0.90, 1.12) | 1.30  (0.83, 2.02) | 1.49  (1.01, 2.21) | 1.03  (0.92, 1.15) | 1.03  (0.93, 1.15) | 0.98  (0.91, 1.05) | 1.03  (0.97, 1.09) |
| Medical/Crime/Family Problems | 1.23  (0.96, 1.57) | 1.20  (0.96, 1.50) | 1.09  (0.97, 1.24) | 0.98  (0.88, 1.10) | 0.99  (0.66, 1.49) | 1.28  (0.86, 1.93) | 1.07  (0.96, 1.20) | 1.14  (1.03, 1.27) | 1.02  (0.95, 1.09) | 1.00  (0.94, 1.06) |
| Life Transitions | **0.77**  **(0.60, 0.98)** | **1.08**  **(0.87, 1.36)** | 0.88  (0.78, 1.00) | 0.99  (0.89, 1.10) | 0.86  (0.57, 1.30) | 0.85  (0.57, 1.27) | 0.96  (0.86, 1.07) | 0.93  (0.83, 1.03) | 0.98  (0.91, 1.05) | 1.01  (0.95, 1.07) |
| Resilience/Personal Strength | 0.89  (0.70, 1.12) | 0.78  (0.63, 0.97) | 0.89  (0.78, 1.00) | 0.90  (0.81, 1.00) | 0.81  (0.53, 1.24) | 0.78  (0.53, 1.15) | 0.93  (0.83, 1.03) | 0.88  (0.79, 0.97) | 0.95  (0.88, 1.02) | 1.01  (0.95, 1.07) |
| Social/Emotional Support | **1.01**  **(0.79, 1.29)** | **0.69**  **(0.55, 0.86)** | 1.00  (0.88, 1.13) | 0.89  (0.80, 1.00) | 1.03  (0.67, 1.56) | 0.76  (0.51, 1.12) | 0.92  (0.82, 1.03) | 0.84  (0.76, 0.94) | 0.99  (0.92, 1.06) | 1.00  (0.94, 1.06) |
| Religiosity | 1.16  (0.92, 1.47) | 0.94  (0.76, 1.17) | 1.10  (0.97, 1.24) | 1.03  (0.92, 1.14) | 0.98  (0.65, 1.48) | 1.37  (0.92, 2.03) | 0.95  (0.85, 1.06) | 1.00  (0.90, 1.11) | 1.01  (0.94, 1.08) | 1.05  (0.99, 1.11) |

PR=Prevalence Ratio; CI=Confidence Interval

Model adjusted for age (measured continuously), employment status, educational attainment, marital status, annual household income, cardiovascular risk, mental health diagnoses, asthma, smoking status, alcohol consumption and physical activity.

Bolded estimates represent significance at p < 0.05 for the type3 test of the interaction term.

^a^Frequent insomnia symptoms defined as either trouble falling asleep or waking up during the night, 15+ days a month.

^b^Higher total sleep score indicates poorer sleep. Total sleep score was calculated as one point for each of the following: very short, short, or long sleep duration (where very short and short sleep were mutually exclusive); frequent insomnia symptoms; and waking up feeling unrested where very short and short sleep were mutually exclusive. The total poor sleep score ranged from 0-3 with a higher score indicating poorer sleep.

Participants rated the safety of their neighborhood at 5, 10 and 15 years of age as unsafe, somewhat safe, or very safe. Neighborhood safety was measured as safe (if all responses indicated safe locations), somewhat safe (if there was a mix of safe and unsafe responses) and unsafe (if there were no safe responses for all three questions at ages 5, 10 and 15).

# **Supplemental Table 8. Prevalence Ratios of Sleep Health by Stressors and Coping Strategies Stratified by Childhood Resources, Study of Environment, Lifestyle, and Fibroids, 2010-2012 (N=1,661)**

| **Sleep Outcomes** | **Very Short Sleep (<6 hours) vs. 7-9 hours** | | **Short Sleep (<7 hours) vs. 7-9 hours** | | **Frequent Insomnia^a^ Symptoms (≥ 15 days per month** | | **Wake up feeling unrested (≥ 4 days per week)** | | **Total Sleep Score^b^** | |  |
| --- | --- | --- | --- | --- | --- | --- | --- | --- | --- | --- | --- |
|  | **PR (95% CI)** | **PR (95% CI)** | **PR (95% CI)** | **PR (95% CI)** | **PR (95% CI)** | **PR (95% CI)** | **PR (95% CI)** | **PR (95% CI)** | **PR (95% CI)** | **PR (95% CI)** |  |
| **Childhood Resources** | **Poor** | **Non-poor** | **Poor** | **Non-poor** | **Poor** | **Non-poor** | **Poor** | **Non-poor** | **Poor** | **Non-poor** |  |
| Emotional Distress | 1.03  (0.83, 1.29) | 1.30  (1.03, 1.63) | 1.16  (1.04, 1.30) | 1.11  (0.99, 1.25) | 2.39  (1.49, 3.83) | 2.13  (1.39, 3.27) | 1.25  (1.12, 1.40) | 1.43  (1.28, 1.59) | 1.07  (1.00, 1.14) | 0.98  (0.92, 1.04) |  |
| Experienced Racism | 1.07  (0.85, 1.34) | 1.10  (0.88, 1.39) | 1.08  (0.96, 1.20) | 1.18  (1.04, 1.32) | 1.18  (0.79, 1.76) | 1.24  (0.82, 1.86) | 1.04  (0.93, 1.15) | 1.04  (0.94, 1.16) | 1.09  (1.02, 1.17) | 1.06  (0.99, 1.12) |  |
| Perceived Racism | 0.97  (0.77, 1.21) | 0.90  (0.71, 1.13) | 1.00  (0.90, 1.12) | 0.99  (0.88, 1.11) | 1.25  (0.84, 1.86) | 1.05  (0.71, 1.56) | 1.08  (0.97, 1.20) | 1.03  (0.93, 1.15) | 0.99  (0.92, 1.06) | 0.99  (0.94, 1.06) |  |
| Financial Strain | 1.34  (1.05, 1.70) | 1.00  (0.79, 1.26) | 1.11  (0.99, 1.24) | 0.96  (0.85, 1.08) | 1.58  (1.02, 2.46) | 1.27  (0.85, 1.91) | 1.05  (0.94, 1.17) | 1.03  (0.92, 1.14) | 1.02  (0.95, 1.10) | 1.00  (0.94, 1.06) |  |
| Medical/Crime/Family Problems | 1.28  (1.02, 1.60) | 1.18  (0.93, 1.49) | 0.98  (0.88, 1.09) | 1.09  (0.97, 1.22) | 1.00  (0.67, 1.48) | 1.32  (0.88, 1.99) | 1.16  (1.04, 1.29) | 1.09  (0.98, 1.21) | 0.97  (0.91, 1.04) | 1.04  (0.98, 1.11) |  |
| Life Transitions | 1.00  (0.80, 1.25) | 0.87  (0.69, 1.10) | 0.95  (0.85, 1.07) | 0.94  (0.83, 1.06) | 0.86  (0.57, 1.28) | 0.87  (0.58, 1.30) | 1.00  (0.90, 1.12) | 0.89  (0.80, 0.99) | **0.93**  **(0.86, 0.99)** | **1.06**  **(0.99, 1.12)** |  |
| Resilience/Personal Strength | 0.83  (0.66, 1.04) | 0.82  (0.65, 1.03) | **0.97**  **(0.87, 1.09)** | **0.83**  **(0.74, 0.93)** | 0.77  (0.51, 1.17) | 0.80  (0.54, 1.20) | 0.93  (0.83, 1.03) | 0.87  (0.78, 0.97) | 1.03  (0.96, 1.10) | 0.95  (0.90, 1.01) |  |
| Social/Emotional Support | | 0.96  (0.77, 1.22) | 0.71  (0.56, 0.89) | 0.99  (0.88, 1.11) | 0.92  (0.82, 1.03) | 1.04  (0.69, 1.56) | 0.75  (0.50, 1.13) | **0.98**  **(0.88, 1.09)** | **0.81**  **(0.73, 0.90)** | 1.00  (0.94, 1.08) | 1.00  (0.94, 1.06) |
| Religiosity | 1.00  (0.80, 1.24) | 1.05  (0.84, 1.32) | 1.06  (0.95, 1.18) | 1.04  (0.92, 1.17) | 1.14  (0.76, 1.70) | 1.20  (0.80, 1.78) | 0.91  (0.82, 1.01) | 1.03  (0.92, 1.14) | 1.04  (0.97, 1.11) | 1.02  (0.96, 1.09) |  |

PR=Prevalence Ratio; CI=Confidence Interval

Model adjusted for age (measured continuously), employment status, educational attainment, marital status, annual household income, cardiovascular risk, mental health diagnoses, asthma, smoking status, alcohol consumption and physical activity.

Bolded estimates represent significance at p < 0.05 for the type3 test of the interaction term.

^a^Frequent insomnia symptoms defined as either trouble falling asleep or waking up during the night, 15+ days a month.

^b^Higher total sleep score indicates poorer sleep. Total sleep score was calculated as one point for each of the following: very short, short, or long sleep duration (where very short and short sleep were mutually exclusive); frequent insomnia symptoms; and waking up feeling unrested where very short and short sleep were mutually exclusive. The total poor sleep score ranged from 0-3 with a higher score indicating poorer sleep.

Participants rated the safety of their neighborhood at 5, 10 and 15 years of age as unsafe, somewhat safe, or very safe. Neighborhood safety was measured as safe (if all responses indicated safe locations), somewhat safe (if there was a mix of safe and unsafe responses) and unsafe (if there were no safe responses for all three questions at ages 5, 10 and 15).

Childhood resources was measured as poor if participants 1) marked yes to “times when you didn’t have enough to eat” and 2) answered low income to “household income while growing up”
